# Low-Fat Diet With Caloric Restriction Reduces White Matter Microglia Activation During Aging

**DOI:** 10.3389/fnmol.2018.00065

**Published:** 2018-03-12

**Authors:** Zhuoran Yin, Divya D. Raj, Wandert Schaafsma, Roel A. van der Heijden, Susanne M. Kooistra, Aaffien C. Reijne, Xiaoming Zhang, Jill Moser, Nieske Brouwer, Peter Heeringa, Chun-Xia Yi, Gertjan van Dijk, Jon D. Laman, Erik W. G. M. Boddeke, Bart J. L. Eggen

**Affiliations:** ^1^Department of Neurology, Tongji Hospital, Tongji Medical College of HUST, Huazhong University of Science and Technology, Wuhan, China; ^2^Department of Neuroscience, Medical Physiology Section, University Medical Center Groningen, University of Groningen, Groningen, Netherlands; ^3^Department of Pathology and Medical Biology, Medical Biology Section, University Medical Center Groningen, University of Groningen, Groningen, Netherlands; ^4^Laboratory of Pediatrics, Systems Medicine of Metabolism and Signaling Section, University Medical Center Groningen, University of Groningen, Groningen, Netherlands; ^5^Systems Biology Centre for Energy Metabolism and Ageing, University of Groningen, Groningen, Netherlands; ^6^Groningen Institute for Evolutionary Life Sciences, Department of Behavioral Neuroscience, University of Groningen, Groningen, Netherlands; ^7^Department of Critical Care, University Medical Center Groningen, University of Groningen, Groningen, Netherlands; ^8^Department of Endocrinology and Metabolism, Academic Medical Center, University of Amsterdam, Amsterdam, Netherlands; ^9^ESRIG Centre for Isotope Research, University of Groningen, Groningen, Netherlands

**Keywords:** aging, high-fat diet, low-fat diet, caloric restriction, physical exercise, microglia, neuroinflammation

## Abstract

Rodent models of both aging and obesity are characterized by inflammation in specific brain regions, notably the corpus callosum, fornix, and hypothalamus. Microglia, the resident macrophages of the central nervous system, are important for brain development, neural support, and homeostasis. However, the effects of diet and lifestyle on microglia during aging are only partly understood. Here, we report alterations in microglia phenotype and functions in different brain regions of mice on a high-fat diet (HFD) or low-fat diet (LFD) during aging and in response to voluntary running wheel exercise. We compared the expression levels of genes involved in immune response, phagocytosis, and metabolism in the hypothalamus of 6-month-old HFD and LFD mice. We also compared the immune response of microglia from HFD or LFD mice to peripheral inflammation induced by intraperitoneal injection of lipopolysaccharide (LPS). Finally, we investigated the effect of diet, physical exercise, and caloric restriction (40% reduction compared to *ad libitum* intake) on microglia in 24-month-old HFD and LFD mice. Changes in diet caused morphological changes in microglia, but did not change the microglia response to LPS-induced systemic inflammation. Expression of phagocytic markers (i.e., Mac-2/Lgals3, Dectin-1/Clec7a, and CD16/CD32) in the white matter microglia of 24-month-old brain was markedly decreased in calorically restricted LFD mice. In conclusion, LFD resulted in reduced activation of microglia, which might be an underlying mechanism for the protective role of caloric restriction during aging-associated decline.

## Introduction

Aging and obesity are worldwide health issues affecting millions of people (Han et al., 2011). Both aging and obesity are linked to chronic low-grade inflammation that is associated with multisystem diseases (Lumeng and Saltiel, 2011; Woods et al., 2012). Aging is considered as a pro-obesogenic factor, associated with age-related metabolic decline, which is characterized by changes in fat distribution, obesity, and insulin resistance (Gabriely et al., 2002). Concurrently, obesity can aggravate age-related decline in physical function and cause frailty (Villareal et al., 2005). A number of obesity-associated comorbidities such as type 2 diabetes (Eckel et al., 2011), hypertension (Kotsis et al., 2010), and cardiovascular disease (Poirier et al., 2006) may ultimately contribute to premature aging and shortened lifespan. Importantly, obesity not only affects the function of peripheral organs, but also influences the CNS. Previous studies reported that obesity is associated with synapse loss (Bocarsly et al., 2015), hypothalamic gliosis, and cognitive deficits (Thaler et al., 2013; Kälin et al., 2015). It was also proposed that obesity and its direct comorbidities act as a facilitator and predictor of neurodegenerative diseases (Cai, 2013; van Dijk et al., 2015). Metabolic dysfunction and obesity are associated with learning and memory impairment in early old age (Sabia et al., 2009; Singh-Manoux et al., 2012). It has been suggested that a HFD promotes the progression of obesity (Golay and Bobbioni, 1997). Thus, investigating the pathological alterations resulting from HFD in the aging brain will help to better estimate the role of obesity in neurodegeneration in the elderly.

The brain has long been viewed as an immune-privileged organ with limited access for peripheral immune signals. However, the differently structured blood–brain barrier in the hypothalamic ARC-ME complex allows more blood-borne signals to enter the brain (Gross, 1992). Microglia, the tissue macrophages of the CNS, play a pivotal role in the chronic inflammation observed in aging (Norden and Godbout, 2013; Galatro et al., 2017a,b) as well as metabolic diseases such as obesity (Kälin et al., 2015). Increased inflammation in the aging brain is most predominant in the white matter, and includes loss of myelinated fibers and malformation of myelin sheaths (Gunning-Dixon et al., 2009). In addition to aging, an HFD induces brain inflammation (Zhang et al., 2008). Young wild-type mice on a short-term HFD showed an increase in microglia numbers and pro-inflammatory factors in ARC-ME areas (Thaler et al., 2012; Gao et al., 2014), even before body weight was affected (Gao et al., 2014). HFD may cause central leptin (Münzberg et al., 2004; Enriori et al., 2007) and insulin resistance (De Souza et al., 2005), which underlies increased food intake, body fat content (Rosenbaum and Leibel, 2010), and adaptive thermogenic responses aimed at maintaining the increased body fat content (Koch et al., 2014). Moreover, this condition in the CNS leads to increased hepatic glucose production (Pocai et al., 2005), and collectively this underlies an increased risk toward obesity, diabetes, and cardiovascular disease (Thaler and Schwartz, 2010). In contrast, a prolonged period of HFD was recently reported to induce mixed pro- and anti-inflammatory responses in the hypothalamus (Baufeld et al., 2016). Summarizing these observations, our understanding of the impact of HFD on inflammation and the role of microglia in different brain regions is incomplete.

Lifestyle, e.g., diet pattern and physical exercise, considerably impacts aging phenotypes (Woods et al., 2012; Akbaraly et al., 2013), and indeed, caloric restriction has a preventive effect on age-related chronic diseases (Heilbronn and Ravussin, 2003) and accelerated aging (Vermeij et al., 2016). For example, MRI experiments show that caloric restriction can attenuate age-related deterioration in white matter (Bendlin et al., 2011). The neuroprotective mechanisms of caloric restriction may include: (1) upregulation of BDNF (Stranahan et al., 2009) and heat shock proteins, such as HSP70 (Yu and Mattson, 1999); (2) increased neuronal resistance to excitotoxic damage (Bruce-Keller et al., 1999); and (3) inhibition of the increase of age-related lipid peroxidation (Sohal and Weindruch, 1996; Sharma and Kaur, 2005). Besides reduced intake of calories, there is also growing evidence suggesting that physical exercise induces loss of adipose tissue, decreases the expression of systemic markers of inflammation (Woods et al., 2012), upregulates the level of BDNF (Stranahan et al., 2009), and increases hippocampal neurogenesis (Van Praag et al., 1999; Van der Borght et al., 2009).

The dynamic communication between microglia and all other cell types in the CNS helps them perform both their immune and non-immune functions under homeostasis. Moreover, a variety of activation states has been described for microglia upon disease and injury, where the context dictates whether the outcome for the CNS is tissue-supportive or detrimental (Li and Barres, 2017). Although the knowledge of microglia responses to CNS disease is rapidly increasing, the function and phenotype of microglia under conditions of caloric restriction and physical exercise are still poorly understood. Hence, the aim of this study was to investigate the effects of diet, aging, caloric restriction, and physical exercise on mouse microglia in a basal and inflammatory state induced by LPS. In different regions of the brain, microglia were characterized according to their gene expression pattern, expression of phagocytosis markers and morphology.

## Materials and Methods

### Mice

All adult mice of different ages were used and kept under specified pathogen-free conditions on a 12-h light/dark cycle and lights switched on at 7:00 am. Animals were sacrificed in the morning, between 9:00 am and 11:00 am. Details on experimental design per mouse strain are described below and in **Table 1** (i.e., animal strain, supplier, age, exercise, and diet). Throughout the experiments, mice were kept under close observation and regularly weighted. All animal work was approved by the Animal Ethics Committee of University of Groningen, and adhered to the European Directive (2010/63/EU) on the protection of animals used for scientific purposes.

**Table 1:** Mouse strain, supplier, group, and treatment information.

Animals			Food intake/exercise/treatment						Age at time of sacrifice				Processing of tissue			Data	
Strain	Sex	Diet	Fat composition	Age at start diet (weeks)	AL	CR	RW	LPS	6 m	9 m	12 m	24 m	Snap frozen	4% PFA fixed	FACS-sorting	Assay	Figs
C57BL/6 OlaHsd, Harlan	Male	HFD	42% fat, ab Diets^®^ 4031.09	4	+				+				+	+		IHC	5
		HFD			+							+	+	+		IHC	5
		HFD					+					+	+	+		IHC	5
		HFD				+						+	+	+		IHC	5
		LFD	6.5% fat, ab Diets^®^ 2141 AM-II	4	+				+				+	+		IHC	5
		LFD			+							+	+	+		IHC	5
		LFD					+					+	+	+		IHC	5
		LFD				+						+	+	+		IHC	5
C57BL/6J, Charles River	Male	HFD	45% fat, Diets^®^ D12451	12	+				+	+	+			+		IHC	1
		LFD	10% fat, Diets^®^ D12450H	6	+				+	+	+			+		IHC	1
C57BL/6J, Charles River	Male	HFD	60% fat, Diets^®^ D12492	12	+			+	+						+	qPCR	2, 3
		HFD			+				+						+	qPCR	2, 3
		LFD	10% fat, Diets^®^ D12450B	6	+			+	+						+	qPCR	2, 3
		LFD			+				+						+	qPCR	2, 3

*HFD, high fat diet; LFD, low fat diet; AL, *ad libitum*; RW, running wheel; CR, caloric restriction; IHC, immunohistochemistry; PFA, paraformaldehyde.*

#### C57BL/6 OlaHsd (Harlan)

C57BL/6 OlaHsd mice were obtained from Harlan and subsequently bred in-house at the University of Groningen.

#### Food Composition and Intake

Male C57BL/6 OlaHsd mice were subjected to both low-fat (LFD; 6.5% fat, ab Diets 2141 AM-II, Supplementary Data Sheet 1) and high-fat (HFD; 42% fat, ab Diets 4031.09, Supplementary Data Sheet 1) diets. The mice were exposed to the different diets and/or restriction from weaning until sacrifice. Weaning was performed at day 28 (day 0 was day of birth), after which mice were individually housed with *ad libitum* access to food and water. For the caloric restriction group, mice were exposed to 40% caloric restriction. The restricted mice received their food 3 h before lights were turned off (7:00 pm); they consumed the food within these 3 h, mostly faster.

#### Wheel Running and Monitoring

Mice had free access to the running wheels. Voluntary wheel-running activity was recorded throughout the lifetime of the mice. The passing of a magnet embedded in the running wheel past the sensor on the cage would signal a wheel revolution. These revolutions were collected continuously and stored in 1 min bins by a Circadian Activity Monitor System (CAMS, by H. M. Cooper and J. A. Cooper, INSERM U846, Department of Chronobiology, Bon, France). Raw data were imported into a custom-made excel macro package (ACTOVIEW, C. K. Mulder, Department of Molecular Neurobiology, Groningen, Netherlands). Initial imports condensed the data to 60-min bins, allowing for easy visual inspection of long-term recordings.

#### C57BL/6J (Charles River)

For the cohorts of C57BL/6J animals from Charles River, the mice were treated as described previously (van der Heijden et al., 2015a). Male C57BL/6J mice at the age of 6 weeks (20 ± 2 g) were individually housed with *ad libitum* access to food and water. After arrival, all mice received a low-fat control diet (LFD; 10% lard; Research Diets Inc., New Brunswick, NJ, United States; D12450HY, Supplementary Data Sheet 1) for 6 weeks after which mice were either switched to a HFD (45% lard; Research Diets; D12451, Supplementary Data Sheet 1) or kept on the low-fat control diet. All animals were kept on their respective diets until time of sacrifice.

### LPS Injection

High-fat diet and LFD mice (6 months) were injected intraperitoneally (i.p.) with 1 mg/kg LPS (Sigma-Aldrich, L4391) or PBS. After 3 h, animals were anesthetized and perfused with 0.9% saline. The brains were sagittally separated and used for two purposes: (1) from one half, microglia were acutely isolated and the RNA extracted (*n* = 5); (2) from the other half, the hypothalamus was dissected and RNA isolated (*n* = 5).

### Acute Isolation of Microglia and Cell Sorting

Microglia were isolated as described in Galatro et al. (2017b) Brain tissue (*n* = 5) was mechanically homogenized in HBSS with 0.6% glucose and 7.5 mM HEPES followed by centrifugation at 220 RCF, 4°C for 10 min. In order to remove myelin, the pellet was re-suspended in a mixture of 22% Percoll (GE Healthcare, 17-0891-01) and 77% myelin gradient buffer (5.6 mM NaH_2_PO_4_.2H_2_O, 20 mM Na_2_HPO_4_.2H_2_O, 140 mM NaCl, 5.4 mM KCl, 11 mM glucose) and 40 mM NaCl, and centrifuged for 20 min at 950 RCF at 4°C. The remaining cell pellet was incubated with phycoerythrin (PE)-coupled rat anti-mouse CD11b (Clone M1/70, eBiosciences), FITC-coupled rat anti-mouse CD45 (Clone 30-F11, eBiosciences). Forward and side scatter parameters were used to identify single cells and the exclusion of DAPI was used to select live cells. Microglia were defined as CD11b^pos^CD45^int^DAPI^neg^ and sorted on a BD FACSAria II (BD Biosciences).

### Primary Microglia Culture and LPS Stimulation

Primary microglia cultures were prepared from brains of postnatal day-1 wild-type male C57Bl/6J mice as previously described (Schaafsma et al., 2015). Briefly, after the removal of the meninges and brain stem, the brains were triturated and washed in medium A (HBSS with 0.6% glucose [D-(+)-glucose solution, Sigma, G8769], 15 mM HEPES buffer (Lonza BE17-737E), 1% penicillin/streptomycin). The minced brains were incubated in 2.5% trypsin at 37°C for 20 min. The enzymatic reaction was stopped by adding trypsin inhibitor medium, followed by washing with medium containing medium A, 10% FCS, and 0.5 μg/ml DNaseI. Cells were triturated using a glass pipette in 25 ml normal medium (Dulbecco’s Modified Eagle Medium, Lonza, BE12-707F, 10% FCS, 1 mM sodium pyruvate, Gibco, 11360-070, and 1% penicillin/streptomycin), and centrifuged at 960 rpm at 12°C for 12 min. The supernatant was removed, and then the pellet was resuspended and plated as 1.5 brains per T75 culture flask in DMEM supplemented with 10% FCS. After 7 days of culture, 5 ml L929 fibroblast-conditioned medium was added to 10 ml culture medium per flask to stimulate microglia proliferation. After around 10 days of culture, astrocytes reached 100% confluence, and microglia were harvested at the 14^th^ day by orbital shaking for 1 h at 150 rpm at 37°C. Microglia were seeded into 3.5 cm 6-well plates, and cultured in the above-mentioned normal medium and medium collected from the mixed glial cultures in a ratio of 1:1 at 37°C with 5% CO_2_. Male and female pups were separated based on anogenital distance. The sex of the pups used for the cultures was confirmed by PCR on genomic DNA using primers for gene loci on X- and Y-chromosomes.

Cultured microglia were stimulated with recombinant mouse leptin (1 μg/ml, R&D, CF 498-OB-01M) for 24 h, followed by 100 ng/ml LPS stimulation for 3 h.

### RNA Isolation and RT-qPCR

RNA from sorted microglia and cultured microglia was extracted using the RNeasy Plus Micro Kit (Qiagen, 74034) and RNA from hypothalamus was extracted using RNeasy Plus Mini Kit (Qiagen, 74134). After RNA isolation, reverse-transcription PCR (RT-PCR) was performed using Applied Biosystems Gene Amp 9700 thermal cycler, and quantitative PCR was performed as previously described (Raj et al., 2014). See primer information in Supplementary Table 1.

### Tissue Sampling for Immunohistochemistry

Animals (details are shown in **Table 1**) were sacrificed and transcardially perfused with saline. Brains were separated, then snap-frozen in liquid nitrogen or fixed in 4% paraformaldehyde (PFA) in PBS fixed for 1 day (details are shown in **Table 1**), dehydrated with 25% sucrose in PBS overnight at 4°C, and then frozen at -50°C. All samples were stored at -80°C.

### Immunohistochemistry

#### PFA-Fixed Tissue

Brain sections (30 μm) from 4% PFA-fixed mouse brains were pre-incubated in 0.3% H_2_O_2_ at room temperature (RT) for 30 min, and then in 10% serum at RT for 1 h. Sections were incubated with the primary antibody at 4°C overnight: Iba1 (1:1000, WAKO, 019-19741) or Mac-2 (1:1000, Cedarlane, CL8942AP). After PBS washing, sections were incubated with the secondary antibody: biotinylated goat anti-rabbit IgG (1:400, Vector Laboratories, BA-1000) or biotinylated rabbit anti-rat IgG (1:400, Vector Laboratories, BA-4001) at RT for 1 h, then with avidin-biotin-peroxidase complex (Vector Laboratories, PK-6100) at RT for 30 min, and finally visualized with 3, 3′-diaminobenzidine (DAB, Sigma, D-5637).

#### Snap-Frozen Tissue

Brain sections (14 μm) from snap-frozen mouse brains were acetone-fixed for 10 min, pre-incubated in Peroxidase Blocking Reagent (DAKO, K4009) for 15 min, and blocked in 5% fetal bovine serum for 30 min (RT). Sections were incubated with primary antibodies at RT for 2 h: Dectin-1 (1:100, AbDSerotec, MCA2289) or CD16/CD32 (1:100, eBioscience, 14-0161-82). Next, sections were incubated with unconjugated rabbit anti-rat IgG (Vector Laboratories, AI-4001) at RT for 1 h. Then sections were incubated with labeled polymer-HRP anti-rabbit (DAKO, K4009) at RT for 30 min. The complex was visualized after 10 min of incubation with 3-amino-9-ethylcarbazole (AEC) substrate-chromogen solution (DAKO, K4009) and counterstained with hematoxylin. The visualization and counterstaining were both done at RT.

### Imaging Analysis

**FIGURE 1:**
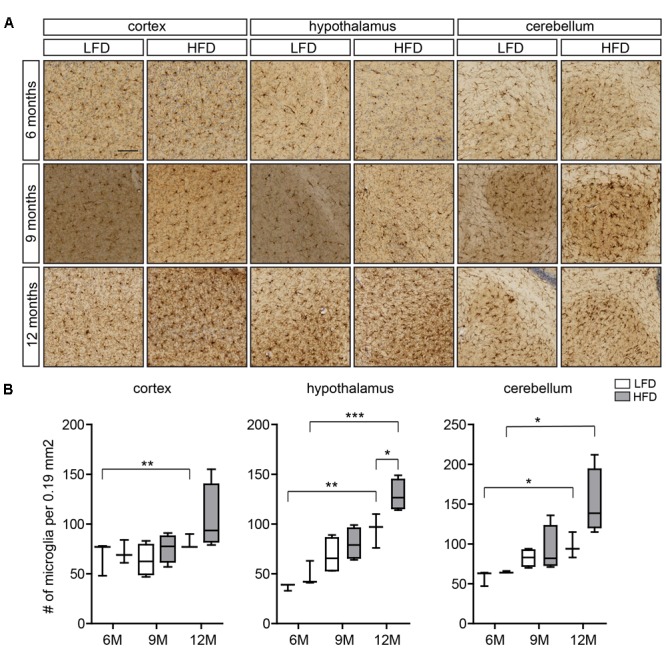
Microglia number in the cortex, hypothalamus, and cerebellum of HFD and LFD mice. **(A)** Representative images of brain sections of HFD and LFD mice at the indicated ages immunostained for Iba1, and counterstained with Cresyl violet. Iba1^+^ microglia in the cortex, hypothalamus, and cerebellum were analyzed. **(B)** The number of microglia was compared between HFD animals and LFD animals at the indicated ages. In both HFD and LFD mice, the number of microglia increased during aging. The number of microglia was significantly increased in the hypothalamus of 12-month-old HFD animals (*n* = 3 mice, gray boxes depict HFD samples, white boxes depict LFD samples, Student’s *t*-test, ^∗^*p* < 0.05, ^∗∗^*p* < 0.01, ^∗∗∗^*p* < 0.001). Scale bar: **(A)** = 100 μm.

Immunohistochemically stained sections were imaged using a Hamamatsu Nanozoomer (Hamamatsu Photonics). The images of AEC-stained sections (**Figure 5**) were analyzed with the positive pixel count algorithm (Imagescope). 5–10 images (20× magnification) from the internal capsule were quantified for each sample (*n* = 4–7). The average number of positive pixels was compared between groups. The images of DAB-stained sections (**Figure 1**) were analyzed using Image J. Microglia cell numbers were determined by binary thresholding in ImageJ followed by ‘analyze particles’ (pixels 10–infinity) and ‘cell count’ (*n* = 3–4 mice/condition).

### Statistical Analysis

Data were analyzed using GraphPad Prism software. For comparison of two different groups, a two-tailed Student’s *t*-test was used. Comparisons of multiple groups were analyzed by one-way analysis of variance (ANOVA) followed by *post hoc* analysis using Bonferroni’s multiple-comparison test. Asterisks indicate: ^∗^*p* < 0.05, ^∗∗^*p* < 0.01, ^∗∗∗^*p* < 0.001.

## Results

### HFD Augments the Aging-Induced Increase in Microglial Cell Number and Morphological Changes

Here we evaluated regional differences in the number of microglia of 6-, 9-, and 12-month-old HFD (45% fat) and LFD (10% fat) mice using Iba1 immunohistochemistry (**Figure 1A**). Microglia numbers were determined in the cortex, hypothalamus, and cerebellum (**Figure 1B**). As described previously, Iba1 reactivity of microglia in the white matter of the CNS increases with age (Raj et al., 2017). In the cortex, no significant differences in the number of microglia between LFD and HFD mice were detected at these respective ages (**Figure 1B**). In the hypothalamus, microglia numbers were significantly higher in both HFD and LFD mice at 12 months of age compared to 6-month-old mice; and at 12 months microglia numbers were significantly increased in HFD compared to LFD mice (**Figure 1B**). In the cerebellum, microglia numbers were significantly increased in 12-month-old mice compared to 6-month-old mice for both HFD and LFD mice (**Figure 1B**).

### The Effect of HFD on LPS-Induced Gene Expression in Total Brain Microglia

**FIGURE 2:**
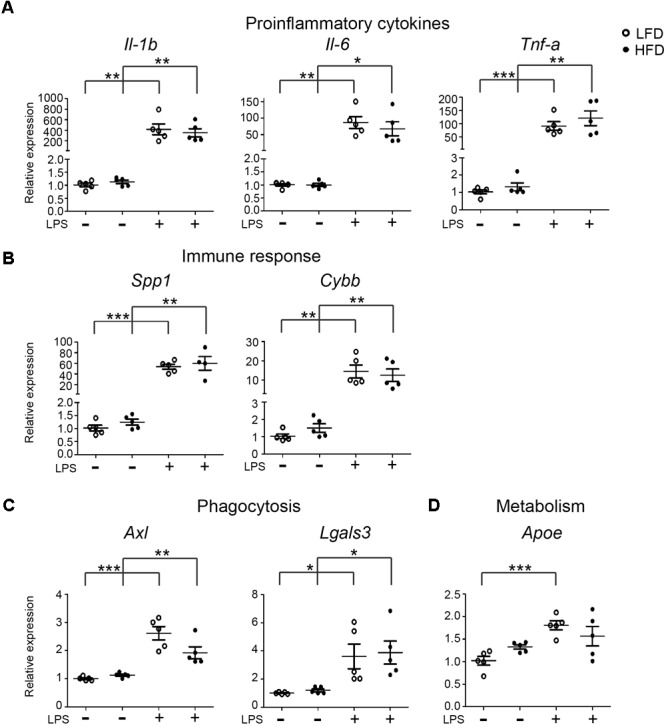
Expression of inflammatory, phagocytic and metabolism genes in LFD and HFD microglia. 6-month-old HFD and LFD animals were i.p. injected with LPS or PBS. Microglia were FACS isolated and RNA was extracted and quantified using RT-qPCR. RNA expression levels were normalized to Hmbs levels as in internal control and the expression levels in PBS-injected LFD mice were set at 1. Gene expression levels were compared between both PBS- and LPS-injected mice and between HFD and LFD animals. **(A)** proinflammatory cytokines (*Il-1*β, *Il-6*, and *Tnf*-α), **(B)** immune response (*Spp1* and *Cybb*), **(C)** phagocytosis (*Axl, Lgals3*), and **(D)** metabolism (*Apoe*) genes were significantly upregulated after LPS injection, but no significant difference between HFD+LPS and LFD+LPS samples was detected. Open circles depict LFD and closed circles depict HFD samples (*n* = 5 mice, mean ± SEM is depicted, Student’s *t*-test, ^∗^*p* < 0.05, ^∗∗^*p* < 0.01, ^∗∗∗^*p* < 0.001).

To assess if HFD affected the microglia response to an inflammatory challenge, microglia were isolated from the brain of 6-month-old LFD and HFD mice, 3 h after an intraperitoneal (i.p.) injection with PBS or LPS (1 mg/kg). The mRNA expression levels of pro-inflammatory, phagocytic, and metabolic genes were determined. The basal expression of pro-inflammatory genes *Il-1β, Tnf-α*, and *Il-6* and other immune-related genes *Spp1* and *Cybb* was not significantly different between HFD and LFD microglia. As expected, i.p. injection with LPS resulted in a significant increase in proinfammatory and immune gene expression. However, this response was not significantly different between HFD and LFD microglia (**Figures 2A,B**). Expression levels of the phagocytic markers *Axl* and *Lgals3* (*Mac-2*) were induced by LPS but not significantly different between LFD and HFD conditions (**Figure 2C**). *Apoe*, a gene involved in lipid metabolism, was induced by LPS, in both LFD and HFD microglia (**Figure 2D**). Collectively, these data indicate that a 6-month HFD did not result in generalized microglial activation or an altered responsiveness to an inflammatory stimulus such as LPS.

### The Effect of HFD on LPS-Induced Gene Expression in Total Hypothalamus

**FIGURE 3:**
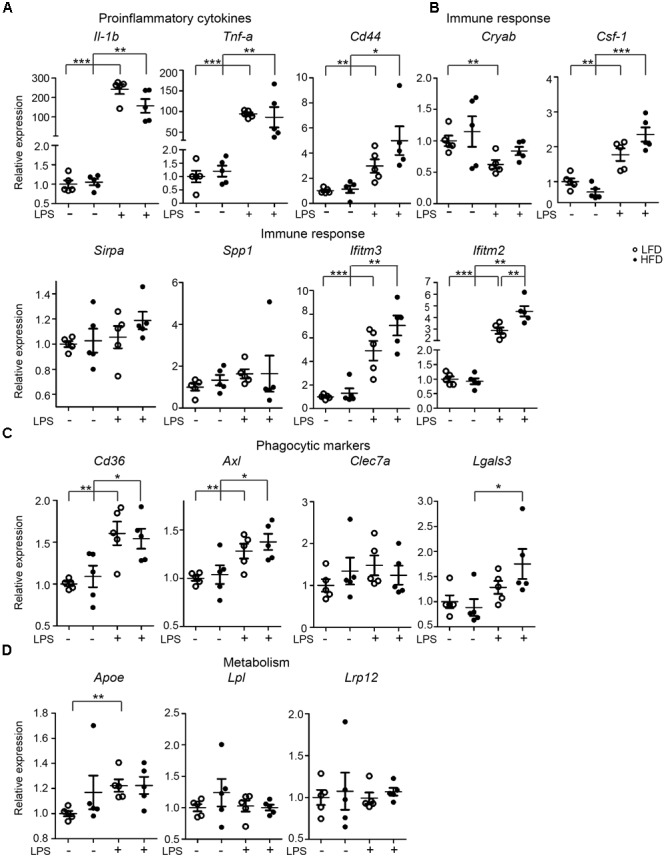
Expression of immune-related genes in the hypothalamus of PBS/LPS-treated LFD and HFD mice. 6-month-old HFD and LFD animals were i.p. injected with LPS or PBS, total RNA was extracted from the hypothalamus and analyzed using RT-qPCR. The gene expression of **(A)** proinflammatory cytokines (*Il-1β* and *Tnf-α*), **(B)** genes relating to immune response (*CD44, Cryab, Sirpa, Spp1, Ifitm3*, and *Ifitm2*), **(C)** phagocytic markers (*Axl, Lgals3, CD36*, and *Clec7a*), and **(D)** genes relating to lipid metabolism (*Apoe, Csf-1, Lpl*, and *Lrp12*) were compared between HFD and LFD animals, but also between samples with or without LPS injection. Open circles depict LFD and closed circles depict HFD samples (*n* = 5 mice, mean ± SEM is depicted, Student’s *t*-test, ^∗^*p* < 0.05, ^∗∗^*p* < 0.01, ^∗∗∗^*p* < 0.001).

In microglia isolated from total mouse brain, no significant changes in LPS-induced inflammatory responses were observed under LFD and HFD conditions. However, it is possible that regional effects of LFD/HFD on microglia were obscured when analyzing total brain microglia. The hypothalamus for example is substantially affected by HFD (Thaler et al., 2012; Gao et al., 2014). Hence, gene-expression levels of microglia-associated genes were determined in the hypothalamus of 6-month-old LFD and HFD mice, which received an i.p. injection of PBS or LPS (1 mg/kg). LPS induced a significant increase in expression of the proinflammatory cytokine genes *Il-1β* and *Tnf-α* in the hypothalamus of both HFD and LFD mice, but their expression was not significantly different between HFD and LFD mice (**Figure 3A**). With the exception of *Ifitm2*, no significant effect of HFD on the expression of several immune-response genes, *CD44, Cryab, Csf-1, Sirpa, Spp1*, and *Ifitm3* was detected. LPS significantly induced *CD44, Csf-1*, and *Ifitm3* expression levels in both LFD and HFD hypothalamus (**Figure 3B**). No substantial differences in the hypothalamus were observed in the expression of phagocytic markers *CD36, Axl, Clec7a* (*Dectin-1*), and *Lgals3* between LFD and HFD hypothalamus was detected. LPS significantly increased expression of *CD36, Axl*, and *Lgals3* in LFD and HFD hypothalamus (**Figure 3C**). No significant effect of HFD on *Apoe, Lpl*, and *Lrp12* was detected (**Figure 3D**). In summary, these data indicate that LPS treatment resulted in increased expression of a range of immune-, phagocytosis-, and metabolism-associated genes in the hypothamalus, but no significant differences were detected between LFD and HFD samples.

### Increase in Leptin Plasma Levels Does Not Induce Hyperreactivity of Microglia

**FIGURE 4:**
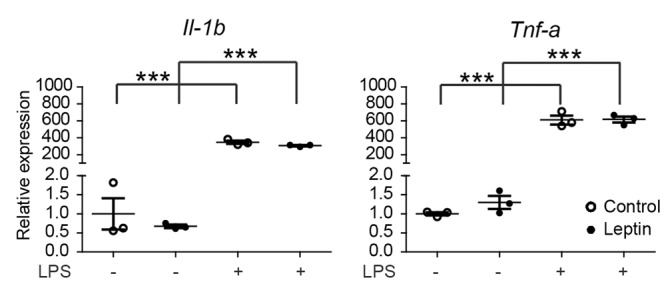
Leptin does not alter microglia responsiveness to LPS. Male primary mouse microglia were incubated with leptin (1 μg/ml) for 24 h, followed by an LPS stimulation (100 ng/ml) for 3 h. Gene expression of proinflammatory cytokines (*Il-1β* and *Tnf-α*) was compared between control and leptin-treated cells with or without LPS stimulation. Leptin treatment had no significant effect on the LPS-induced expression of proinflammatory cytokines. Open circles depict untreated and closed circles depict leptin-treated microglia (*n* = 3 microglia cultures, mean ± SEM is depicted, one-way ANOVA with Bonferroni *post hoc* comparison, ^∗∗∗^*p* < 0.001).

The effects of HFD or LFD on serum leptin levels and body weight in the C57BL/6J mouse cohort were reported earlier (van der Heijden et al., 2015a,b). In mice on a HFD (45% fat), leptin plasma levels strongly increased from 10 to 40 ng/ml, peaked around 28 weeks and remained elevated. Previous data indicated that *in vitro* pretreatment of rat microglia with leptin for 24 h, was capable of potentiating a subsequent LPS response, resulting in approximately 2-fold higher Il-1β release (Pinteaux et al., 2007; Lafrance et al., 2010). However, a concomitant increase in *Il-1β* mRNA was not detected (Lafrance et al., 2010). As described above, we did not observe an effect on LPS responsiveness *in vivo*. Therefore, we next evaluated if leptin (1 μg/ml) altered the inflammatory response of male primary microglia *in vitro*. In primary microglia, LPS induced a significant upregulation of the pro-inflammatory genes *Il-1β* and *Tnf-α*. However, in agreement with the data from rat microglia (Lafrance et al., 2010), pretreatment with leptin for 24 h did not alter the microglia response to LPS at the mRNA level (**Figure 4**). These data show that leptin preconditioning of microglia *in vitro* did not result in increased inflammatory gene expression nor in enhanced LPS sensitivity, which is in contrast to aging, where this microglial hyperreactivity was observed (Henry et al., 2009).

### Effects of Running-Wheel Exercise and Caloric Restriction on White Matter Inflammation

**FIGURE 5:**
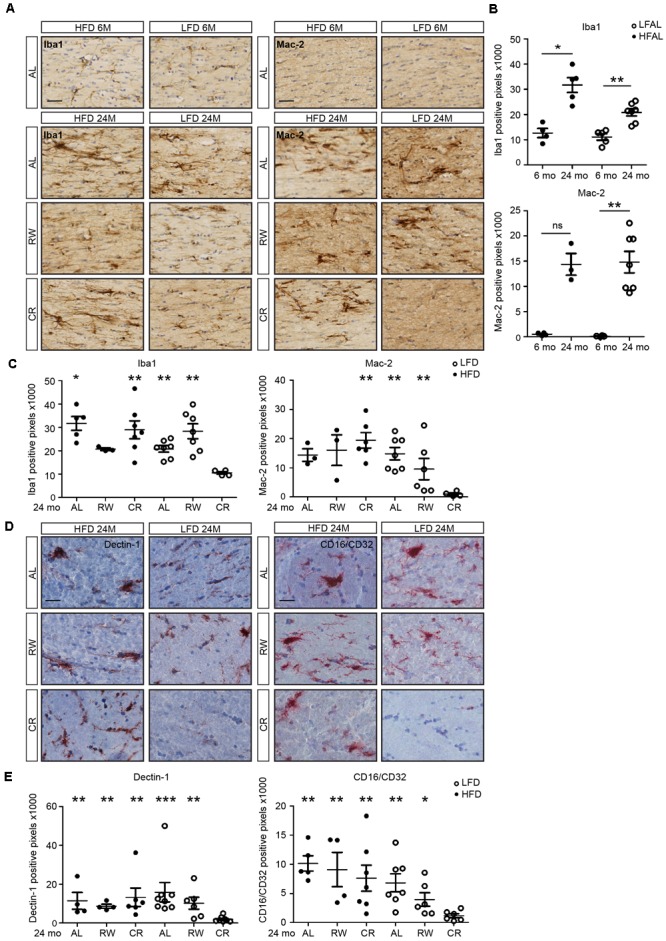
Low-fat diet with caloric restriction reduces white matter microglia activation during aging. **(A)** Brain sections of 6- and 24-month-old mice on different diets and regimes were immunostained for Iba1 and Mac-2, and counterstained with cresyl violet. LFAL, low-fat diet *ad libitum*; HFAL, high-fat diet *ad libitum*; LFRW, low-fat diet with running wheel; HFRW, high-fat diet with running wheel; LFCR, low-fat diet with caloric restriction; HFCR, high-fat diet with caloric restriction. **(B)** Quantification of conditions depicted in the top panel of **(A)**. The expression of Iba1 and Mac-2 increased during aging in both HFAL and LFAL groups (*n* = 3–7 mice, mean ± SEM is depicted, Mann–Whitney test, ns, not significant, ^∗^*p* < 0.05, ^∗∗^*p* < 0.01). **(C)** Quantification of conditions depicted in the bottom panel of **(A)**. Compared to the LFCR group, the expression of Iba1 was significantly higher in HFAL, HFCR, LFAL, and LFRW mice. Mac-2 was significantly higher expressed in HFCR, LFAL, and LFRW mice (*n* = 3–7, Mean ± SEM, Mann–Whitney test, ^∗^*p* < 0.05, ^∗∗^*p* < 0.01). **(D)** Tissues from animals on different diets and regimes (LFAL, HFAL, LFRW, HFRW, LFCR, and HFCR) were immunostained for Dectin 1 and CD16/CD32 and counterstained with hematoxylin. **(E)** Dectin 1 and CD16/CD32 were significantly lower expressed in LFCR mice exclusively (*n* = 4–7 mice, mean ± SEM is depicted, Mann–Whitney test, ^∗^*p* < 0.05, ^∗∗^*p* < 0.01, ^∗∗∗^*p* < 0.001). Scale bar: **(A,D)** = 40 μm.

Increased inflammation in the aging human brain is most predominantly observed in the white matter (Gunning-Dixon et al., 2009). However, the effect of long-term diet pattern and exercise on the aging brain and white matter inflammation has not been assessed. We therefore evaluated microglia numbers and activation in white matter regions in 6- and 24-month-old LFD and HFD mice. In addition, 24-month-old mice were subjected either to a lifelong voluntary running wheel paradigm or lifelong caloric restriction (**Figure 5**). At the age of 24 months, both HFD and LFD mice showed increased expression of Iba1. Clustering of microglia was also observed in fimbriae (**Figures 5A,B**). Remarkably, in LFD mice, caloric restriction completely prevented this increase in Iba1 expression, whereas in HFD mice, caloric restriction had little effect (**Figure 5C**). Microglial expression of the phagocytic marker Mac-2 (Lgals3) in the fimbria was increased in both 24-month-old HFD and LFD mice when compared to 6-month-old mice (**Figures 5A,B**). Similar to Iba1, the expression of the phagocytic marker Mac-2 was completely absent in 24-month-old LFD mice with caloric restriction, indicating that LFD plus caloric restriction reduces microglia activation (**Figure 5C**). In another prominent white-matter brain region, the internal capsule, we quantified the expression of phagocytic markers Dectin-1 and CD16/CD32 in 24-month-old HFD- and LFD mice. These mice either had access to a running wheel or were subjected to caloric restriction (**Figures 5D,E**). Remarkably, significant expression of Dectin-1 and CD16/CD32 was observed in the white matter bundle in all experimental groups except in caloric-restricted LFD mice (**Figures 5D,E**).

## Discussion

In this study, we observed a marked effect of aging on microglia numbers in the hypothalamus and cerebellum. In HFD mice, the cell number of microglia increased compared to their age-matched LFD littermates, especially at the age of 9 and 12 months. These results are in accordance with earlier studies. An increase in microglia cell number has been observed previously in the hypothalamus of mice subjected to 20-week HFD (Valdearcos et al., 2014; Baufeld et al., 2016). The expression of inflammatory genes increased significantly in the cerebellum after a 16-week HFD, while those genes in cortex remained unchanged (Guillemot-Legris et al., 2016).

Previous data has indicated that neuroinflammation can be influenced by caloric intake, where not only fat content, but also the source of the fat and carbohydrate content have been reported to influence microglia in the hypothalamus (Maric et al., 2014; Valdearcos et al., 2014; André et al., 2017; Gao et al., 2017). In particular, saturated fatty acids have been connected with microglia-induced inflammation (Valdearcos et al., 2014). More recently, it was shown that carbohydrates are mainly responsible for microgliosis in the hypothalamus of mice on a diet with high caloric content (Gao et al., 2017). However, the fact that we observed increased microglia numbers in mice subjected to caloric restriction in combination with HFD suggests that under our experimental conditions, it is mainly the fat content and/or composition that dictated microglia activation.

Basal expression levels of pro-inflammatory, phagocytosis, immune response, and energy metabolism markers did not differ in microglia isolated from the total brain tissue of HFD and LFD mice. Furthermore, microglial responsiveness to LPS did not differ between HFD and LFD mice, in agreement with earlier results (Baufeld et al., 2016). In rats on an 8-week high-saturated-fat diet, hypothalamic inflammation markers were increased but no effect of diet on the change in core body temperature after a peripheral LPS challenge was observed (Maric et al., 2014).

Previous experiments in HFD mice showed an increased expression of pro-inflammatory cytokines and activation of NF-κB in the hypothalamus (De Souza et al., 2005). Microglia have been proposed to be important players in this inflammatory cascade, perpetuating hypothalamic injury (Zhang et al., 2008; Milanski et al., 2009; Posey et al., 2009; Thaler et al., 2012). Inflammation in the hypothalamus disrupts feeding-related pathways, and promotes insulin and leptin resistance, thereby favoring an elevated body weight (De Souza et al., 2005; Posey et al., 2009; Spencer and Tilbrook, 2011). However, the exact role of microglia in HFD-induced neuroinflammation is still poorly understood. A recent study showed that HFD induced an acute inflammatory response in the hypothalamus after 3 days, but this increased expression of proinflammatory genes was no longer observed after 4 and 8 weeks. In contrast, at these time points a more pronounced anti-inflammatory gene expression profile was observed (Baufeld et al., 2016). However, it has been clearly demonstrated that microglia play critical roles in neuroinflammation in response to HFD. Depletion of microglia resulted in reduced hypothalamic inflammation (Valdearcos et al., 2014) and a similar effect was seen when proliferation of microglia was inhibited (André et al., 2017).

The detrimental effect of HFD on the brain predominantly occurs in the hypothalamus. The hypothalamus has an important role in feeding behavior and neuroendocrine/autonomic outflow, where leptin and insulin play key roles in regulating hunger signals and energy expenditure, mainly by acting through neuronal subpopulations in the arcuate nucleus (Yi et al., 2012). In the hypothalamus, the expression of most genes involved in pro-inflammatory cytokine signaling, immune response, phagocytosis, and metabolism in HFD and LFD mice were not significantly different. However, a significant difference was observed in the expression of interferon-induced transmembrane protein 2 (*Ifitm2*), where HFD mice showed a significant higher expression compared to LFD mice. In addition, the expression of *Ifitm3* showed a strong tendency to being increased in HFD mice compared to LFD mice after LPS injection, although the difference did not reach statistical significance. Ifitm proteins are important players in the type I interferon antiviral response. Deletion of *Ifitm* genes in mice results in an obese phenotype and metabolic dysfunction. The microglia in the hypothalamus of IfitmDel mice have an activated phenotype and an exaggerated pro-inflammatory response to Poly I:C, a viral mimetic and TLR3 agonist (Wee et al., 2015).

Leptin, a satiety hormone secreted by adipose tissue, is essential in regulating body weight and can enter the brain (Rivest, 2002). Since leptin is a prominent factor in the detrimental effects of HFD (Lin et al., 2000), the effect of leptin on microglia was analyzed both *in vivo* and *in vitro*. We observed that high peripheral levels of leptin in HFD mice were not accompanied by an altered responsiveness of microglia to a systemic inflammatory stimulus in comparison to microglia from LFD mice. Previous studies reported that in obesity, the ability of leptin to cross the blood–brain barrier is reduced (Burguera et al., 2000; Banks et al., 2006). In order to test the effect of leptin on microglia directly, we pretreated primary microglia with leptin, which did not change basal inflammatory gene-expression levels or the response to an LPS challenge. In summary, neither HFD nor leptin induced changes in the inflammatory responses of microglia in aging mice.

Although no major differences were observed in cytokine expression levels in microglia isolated from total brain tissue and hypothalamic tissue from HFD and LFD mice, we observed very profound differences in neuroinflammation and phagocytic markers in prominent white matter bundles between HFD and LFD mice at different ages in combination with caloric restriction. Previous studies showed that dietary intervention, such as caloric restriction, slows down the aging process (Johnson et al., 2006; Colman et al., 2014). Indeed a lifelong reduction in caloric intake reduces the oxidative damage in the brain (Mattson et al., 2001), preserves long-term potentiation of synaptic transmission (Hori et al., 1992), and ameliorates cognitive decline (Pitsikas and Algeri, 1992). In peripheral nerves, caloric restriction maintains the nerve structure, improves nerve function, and attenuates the age-associated reduction of myelin proteins and widening of the nodes of Ranvier (Rangaraju et al., 2009). The underlying mechanisms might be the expression of protein chaperones and activation of the autophagy-lysosomal pathway (Rangaraju et al., 2009). Here, the reduction of phagocytic markers in white matter microglia of LFD with caloric restriction might reflect decreased axonal stress in the CNS. However, the pathology of axons at LFD vs. HFD combined with caloric restriction conditions has not been examined yet. Physical exercise, which was provided by a running wheel in our study, reduced the risk of cardiovascular disease, type 2 diabetes, obesity, stroke, and is protective against age-related cognitive decline (McKee et al., 2014). In our study, the expression of Mac-2 and Dectin-1 remained high in the running-wheel HFD group, only the expression of CD16/CD32 was partially decreased in LFD mice in the running-wheel experiment. However, the expression of CD16/CD32 was still significantly higher in HFD mice than in LFD mice with caloric restriction. Although previous studies reported that physical exercise plays an equivalent role in terms of energy balance (Redman et al., 2007) and regulating insulin resistance (Coker et al., 2009), our data suggest that in mice, caloric restriction might be a more effective intervention in protecting white matter structures than physical exercise as expression of markers of microglia activation and phagocytosis (CD16/CD32, Mac-2, and Dectin-1) was notably absent in the white matter of caloric restriction LFD mice.

In this study, we show that HFD increased the number of microglia in the hypothalamus and both number and soma size of microglia were increased in the cerebellum during aging in HFD mice. Under basal- or LPS-induced inflammatory conditions, gene expression analysis of the total brain microglia population or hypothalamus tissue showed similar findings in HFD and LFD mice. Caloric restriction in LFD mice prevented the increased expression of phagocytic markers in white matter microglia with aging, and this protective effect of caloric restriction was not observed in HFD mice. Because running wheel access did not affect white matter microglia activation in either diet, dietary fat as well as caloric content may play an important role in the inflammatory process in brain aging.

## Author Contributions

ZY and DR designed and conducted the RNA experiments. ZY conducted the immunohistochemistry experiments and finalized the figures. ZY and WS analyzed the data and wrote the manuscript. RvdH, AR, JM, PH, and GvD provided the animals. XZ and WS conducted the *in vitro* leptin experiments. NB and C-XY provided technical support. JL, SK, EB, and BE supervised, wrote the manuscript, and provided funding for the study.

## Conflict of Interest Statement

The authors declare that the research was conducted in the absence of any commercial or financial relationships that could be construed as a potential conflict of interest.

## Abbreviations

ARC-ME: arcuate nucleus-median eminence
BDNF: brain-derived neurotrophic factor
CNS: central nervous system
FCS: fetal calf serum
FITC: fluorescein isothiocyanate
HFD: high-fat diet
i.p.: intraperitoneal
LFD: low-fat diet
LPS: lipopolysaccharide
MRI: magnetic resonance imaging
PBS: phosphate buffered saline
PE: phycoerythrin
RT-PCR: reverse transcription PCR
