# Supplementary material for: Low-Fat Diet With Caloric Restriction Reduces White Matter Microglia Activation During Aging
Source: Front Mol Neurosci. 2018 Mar 12;11:65. doi: 10.3389/fnmol.2018.00065 (PMC5857900; doi:10.3389/fnmol.2018.00065)
Supplement: Supplementary file 1 [file Table_1.docx]

**Supplementary Table 1** qPCR primer information

| Gene name | Accession No. | Forward primer 5’-3’ | Reverse primer 5’-3’ |
| --- | --- | --- | --- |
| *Apoe* | NM_000041 | GTTGCTGGTCACATTCCTGG | CTTCAACTCCTTCATGGTCTCG |
| *Axl* | NM_001699 | GTTTGGAGCTGTGATGGAAGGC | CGCTTCACTCAGGAAATCCTCC |
| *Cd36* | NM_001159558 | GATGTGGAACCCATAACTGGA | AGGTACAATGTAAGGTCTCTTCAG |
| *Cd44* | NM_009851.2 | GCAGCAGCTACCCAGCAGGA | GGGCCGAAGCAGTTGTCCCT |
| *Clec7a* | NM_020008 | CCCAACTCGTTTCAAGTCAG | AGACCTCTGATCCATGAATCC |
| *Cryab* | NM_009964 | TGATTGAGGTCCACGGCAAG | TGAAGCCATGTTCGTCCTGG |
| *Csf-1r* | NM_001037859.2 | CAAGATCTGGACAAAGAGGCCA | CCGGTGGATGCAGTTTTTAGAAG |
| *Cybb* | NM_007807 | GCCAGTGTGTCGAAATCTGC | AATTGTGTGGATGGCGGTGT |
| *Ifitm2* | NM_030694.1 | AAGCCTCGACCCTTTACCCT | GCCACCCCGTGCACTTTAT |
| *Ifitm3* | NM_025378 | CCGTGAAGTCTAGGGATCGG | CTTAGCAGTGGAGGCGTAGG |
| *Il-1a* | NM_008361 | CCCAAAAGATGAAGGGCTGC | TGATACTGCCTGCCTGAAGC |
| *Il-6* | NM_031168 | ACAACCACGGCCTTCCCTACTT | CACGATTTCCCAGAGAACATGTG |
| *Lgals3* | NM_010705.3 | CAGGATTGTTCTAGATTTCAGGAG | TGTTGTTCTCATTGAAGCGG |
| *Lpl* | NM_008509 | GAATCGCTGTAACAATCTGGG | GGTAATGGAACACTTTGTAGGG |
| *Lrp12* | NM_172814.3 | ACAAAAGAGTCTCAGCGGCG | AGCACCATTTCCGTACACCC |
| *Spp1* | NM_009263 | AGCAAGAAACTCTTCCAAGCA | CATCCGAGTCCACAGAATCC |
| *Sry* | NM_011564.1 | GCATTTATGGTGTGGTCC | CCAGTCTTGCCTGTATGTGA |
| *Tnf-α* | NM_013693 | TCTTCTGTCTACTGAACTTCGG | AAGATGATCTGAGTGTGAGGG |
| *Xist* | NR_001463.3 | GCTTTGTTTCAGTTTCTCTGG | ATTCTGGACCTATTGGGA |
