# Supplementary material for: Low-Fat Diet With Caloric Restriction Reduces White Matter Microglia Activation During Aging
Source: Front Mol Neurosci. 2018 Mar 12;11:65. doi: 10.3389/fnmol.2018.00065 (PMC5857900; doi:10.3389/fnmol.2018.00065)
Supplement: Supplementary file 2 [file Data_Sheet_1.pdf]

Open formula purified diets for lab animals

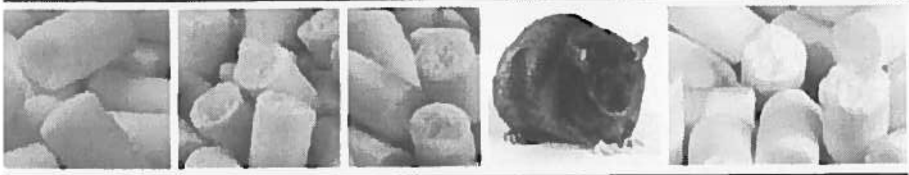

OpenSource DIETS®

Product Data - D12450H

Report ▶ Repeat ▶ Revise

Description
Rodent Diet with 10% kcal% fat.

Used in Research
Obesity
Diabetes

Packaging
Product is packed in 12.5 kg box. Each box is identified with the product name, description, lot number and expiration date.

Lead Time
5-7 business days.

Gamma-Irradiation
Yes. Add 10 days to delivery time.

Form
Pellet, Powder, Liquid

Shelf Life
Most diets require storage in a cool dry environment. Stored correctly they should last 6 months.

Control Diets
Used as a control diet for D12451

Sucrose Content
D12451 Match
17% Sucrose

Formula

Product #D12450H		gm%	kcal%
Protein		19.2	20
Carbohydrate		67.3	70
Fat		4.3	10
	Total		100
	kcal/gm	3.85	

Ingredient	gm	kcal
Casein, 30 Mesh	200	800
L-Cystine	3	12
Corn Starch	452.2	1808.8
Maltodextrin 10	75	300
Sucrose	172.8	691.2
Cellulose, BW200	50	0
Soybean Oil	25	225
Lard*	20	180
Mineral Mix S10026	10	0
DiCalcium Phosphate	13	0
Calcium Carbonate	5.5	0
Potassium Citrate, 1 H2O	16.5	0
Vitamin Mix V10001	10	40
Choline Bitartrate	2	0
FD&C Yellow Dye #5	0.04	0
FD&C Red Dye #40	0.01	0
**Total**	**1055.05**	**4057**

*Typical analysis of cholesterol in lard = 0.72 mg/gram.
Cholesterol (mg)/4057 kcal = 54.4
Cholesterol (mg)/kg = 51.6

RESEARCH DIETS INC.
www.ResearchDiets.com
Where NutriPhenomics Begins

Research Diets, Inc.
20 Jules Lane
New Brunswick, NJ 08901 USA
Tel: 732.247.2390
Fax: 732.247.2340
info@researchdiets.com

© 2012 Research Diets, Inc All rights reserved. D12450H-2-13

Open formula purified diets for lab animals

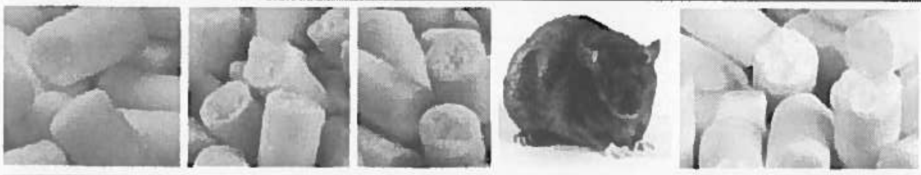

Product Data - D12450B

OpenSource DIETS®

Report ▶ Repeat ▶ Revise

Description
Rodent Diet with 10% kcal% fat.

Used in Research
Obesity
Diabetes

Packaging
Product is packed in 12.5 kg box. Each box is identified with the product name, description, lot number and expiration date.

Lead Time
IN-STOCK.
Ready for next day shipment.

Gamma-Irradiation
Yes. Add 10 days to delivery time.

Form
Pellet, Powder, Liquid

Shelf Life
Most diets require storage in a cool dry environment. Stored correctly they should last 6 months.

Control Diets
Used as a control diet for D12451 and D12492

Sucrose Content
35% Sucrose
See D12450J, K, H for other options.

Formula

Product #D12450B	gm%	kcal%
Protein	19.2	20
Carbohydrate	67.3	70
Fat	4.3	10
Total		100
kcal/gm	3.85	

Ingredient	gm	kcal
Casein, 30 Mesh	200	800
L-Cystine	3	12
Corn Starch	315	1260
Maltodextrin 10	35	140
Sucrose	350	1400
Cellulose, BW200	50	0
Soybean Oil	25	225
Lard*	20	180
Mineral Mix S10026	10	0
DiCalcium Phosphate	13	0
Calcium Carbonate	5.5	0
Potassium Citrate, 1 H2O	16.5	0
Vitamin Mix V10001	10	40
Choline Bitartrate	2	0
FD&C Yellow Dye #5	0.05	0
**Total**	**1055.05**	**4057**

Formulated by E. A. Ulman, Ph.D., Research Diets, Inc., 8/26/98 and 3/11/99.

*Typical analysis of cholesterol in lard = 0.72 mg/gram.
Cholesterol (mg)/4057 kcal = 54.4
Cholesterol (mg)/kg = 51.6

RESEARCH DIETS INC.
www.ResearchDiets.com
Where NutriPhenomics Begins

Research Diets, Inc.
20 Jules Lane
New Brunswick, NJ 08901 USA
Tel: 732.247.2390
Fax: 732.247.2340
info@researchdiets.com

© 2006 Research Diets, Inc. All rights reserved. D12450B–2-13

D12450B, D12451, D12492 The "Original High Fat Diets"

1. Van Heek M, et al. Diet-induced Obese Mice Develop Peripheral, but not Central Resistance to Leptin. J. Clin. Invest. 1997; 99:385-390.
2. Taheri S, et al. Distribution and quantification of immunoreactive orexin A in rat tissues. FEBS Lett. 1999 Aug 20; 457(1):157-61.
3. Nonogaki K, et al. Leptin-independent hyperphagia and type 2 diabetes in mice with a mutated serotonin 5-HT2C receptor gene. Nature Medicine 1998; 4:1152-1156.
4. El-Haschimi K, et al. Two defects contribute to hypothalamic leptin resistance in mice with diet-induced obesity. J.Clin.Invest. 2000; 105:1827-1832.
5. Bush EN, et al.(Metabolic Disease Research, Abbott Laboratories) Adiposity, Leptin Resistance, Hyperrphagia, Hyperglycemia, Glucose Intolerance and Insulin Resistance in C57BL/6J Mice Fed High Fat Diets.Endocrine SocietyAnnual Meeting 2001, Poster Session.
6. Shapiro ME, et al. (Metabolic Disease Research, Abbott Laboratories) Effects of Treatment of C57BL/6j Mice Fed High vs. Low Fat Diets with Metformin or Rosiglitazone on Adiposity, Food Intake, Hyperglycemia and Insulin Resistance. Endocrine Society Annual Meeting 2001, Poster Session.
7. Masuzaki H, Paterson J, Shinyama H, Morton NM, Mullins JJ, Seckl JR, Flier JS. A Transgenic Model of Visceral Obesity and the Metabolic Syndrome. Science. 2001; 294:2166-2170.
8. Razani B, Combs TP, Wang XB, Frank PG, Park DS, Russell RG, Li M, Tang B, Jelicks LA, Scherer PE, Lisanti MP. Caveolin-1 deficient mice are lean, resistant to diet-induced obesity, and show hyper-triglyceridemia with adipocyte abnormalities. J. Biol. Chem. 2001; (in press).
9. Valet P, Grujic D, Wade J, Ito M, Zingaretti MC, Soloveva V, Ross SR, Graves RA, Cinti S, Lafontan M, and Lowell BB. Expression of human alpha 2-adrenergic receptors in adipose tissue of beta 3-adrenergic receptor-deficient mice promotes diet-induced obesity. J.Biol.Chem. 275: 34797-34802, 2000.
10. Vidal-Puig AJ, Grujic D, Zhang CY, Hagen T, Boss O, Ido Y, Szczepanik A, Wade J, Mootha V, Cortright R, Muoio DM, and Lowell BB. Energy metabolism in uncoupling protein 3 gene knockout mice. J.Biol.Chem. 275: 16258-16266, 2000.
11. Ziotopoulou M, Mantzoros CS, Hileman SM, & Flier JS. Differential expression of hypothalamic neuropeptides in the early phase of diet-induced obesity in mice. Am. J. Physiol. Endocrinol. Metab. 279:E383-E845, 2000.
12. Scrocchi LA & Drucker DJ. Effects of Aging and a High Fat Diet on Body Weight and Glucose Tolerance in Glucagon-Like Peptide-1 Receptor -/- Mice. Endocrinology 139:3127-3132, 1998.
13. Ghibaudi L, Cook J, Farley C, Van Heek M, & Hwa J.Fat Intake Affects Adiposity, Comorbidity Factors, and Energy Metabolism of Sprague-Dawley Rats. Obes. Res. 10:956-963. 2002
14. Dube MG, Beretta E, Dhillon H, Ueno N, Kalra PS & Kalra SP. Central Leptin Gene Therapy Blocks High-Fat Diet-Induced Weight Gain, Hyperleptinemeia, and Hyperinsulinemia. Diabetes 51:1729-1736, 2001.
15. Tang H, Vasselli JR, Wu EX, Boozer CN, & Gallagher D. High-Resolution Magnetic Resonance Imaging Tracks Changes in Organ and Tissue Mass in Obese and Aging Rats. Am J Physiol (Regulatory Integrative Comp Physiol) 282:R890-R899, 2002.
16. Bowen H, Mitchell TD, & Harris RBS (Dept. of Foods and Nutr, U of Georgia). Method of Leptin Dosing, Strain, and Group Housing Influence Leptin Sensitivity in High-Fet-Fed Weanling Mice. AJP-Regul Integr Comp Physiol 284:R87-R100, 2003.
17. Harris RBS, Mitchell TD, & Hebert S (Pennington Biomedical Research Center, Baton Rouge, LA). Leptin-Induced Changes in Body Composition in High Fat-Fed Mice. AJPRegul Integr Comp Physiol 284:R87-R100, 2003.
18. Anini, Y. and Brubaker, P. L. Role of Leptin in the Regulation of Glucagon-Like Peptide-1 Secretion. Diabetes 52:252-259, 2003.
19. Bluher, S., Responsiveness to Peripherally Administered Melanocortins in Lean and Obese Mice. Diabetes 53:82-90, 2004.
20. Brunengraber, D. Z., et al. Influence of diet on the modeling of adipose tissue triglycerides during growth. Am J Physiol Endocrinol Metab 285: E917-E925, 2003.
21. Challis, B. G., et al. Mice lacking pro-opiomelanocortin are sensitive to high-fat feeding but respond normally to the acute anorectic effects of peptide-YY3-36. PNAS 101: 13: 4695-4700, 2004.
22. Cohen, A. W., B. Razani, X. B. Wang, T. P. Combs, T. M. Williams, P. E. Scherer, and M. P. Lisanti. Caveolin-1-deficient mice show insulin resistance and defective insulin receptor protein expression in adipose tissue. Am J Physiol Cell Physiol 285: C222-C235, 2003.
23. Combs, T. P., et al. A Transgenic Mouse with a Deletion in the Collagenous Domain of Adiponectin Displays Elevated Circulating Adiponectin and Improved Insulin Sensitivity. Endocrinology 145:367-383, 2004.
24. Conarello, S. L., et al. Mice lacking dipeptidyl peptidase IV are protected against obesity and insulin resistance. PNAS, 100:11: 6825-6830, 2003.
25. Dhar, M. S., et al. Mice Heterozygous for Atp10c, a Putative Amphipath, Represent a Novel Model of Obesity and Type 2 Diabetes. J. Nutr. 134: 799-805, 2004.
26. El-Haschimi, K., S. D. Dufresne, M. F. Hirshman, J. S. Flier, L. J. Goodyear, and C. Bjørbæk. Insulin Resistance and Lipodystrophy in Mice Lacking Ribosomal S6 Kinase 2. Diabetes 52:1340-1346, 2003.
27. Felipe, F., M. L. Bonet, J. Ribot, and A. Palou. Modulation of Resistin Expression by Retinoic Acid and Vitamin A Status. Diabetes 53:882-889, 2004.
28. Felipe, F., M.L. Bonet, J. Ribot and A. Palou. Up-regulation of muscle uncoupling protein 3 gene expression in mice following high fat diet, dietary vitamin A supplementation and acute retinoic acid-treatment. International Journal of Obesity 27: 60-69, 2003.
29. Fu, J., et al. Oleylethanolamide regulates feeding and body weight through activation of the nuclear receptor PPAR. Nature 425:90-93, 2003.
30. Gavrilova, O., et al. Liver Peroxisome Proliferator-activated Receptor Contributes to Hepatic Steatosis, Triglyceride Clearance, and Regulation of Body Fat Mass. The Journal of Biological Chemistry 278:36:34268-34276, 2003.
31. Haluzík, M., O. Gavrilova and D. LeRoith. Peroxisome Proliferator-Activated Receptor-Deficiency Does Not Alter Insulin Sensitivity in Mice Maintained on Regular or High-Fat Diet: Hyperinsulinemic-Euglycemic Clamp Studies. Endocrinology 145:1662-1667, 2004.
32. Hancock, A.A. et al. Antiobesity effects of A-331440, a novel non-imidazole histamine H3 receptor antagonist. European Journal of Pharmacology 487:183-197, 2004.
33. Hennige, A. M., et al. Upregulation of insulin receptor substrate-2 in pancreatic b cells prevents diabetes. J. Clin. Invest. 112:1521-1532, 2003.34.
34. Hildebrandt, A. L., D. M. Kelly-Sullivan, S. C. Black. Validation of a high-resolution X-ray computed tomography system to measure murine adipose tissue depot mass in situ and longitudinally. Journal of Pharmacological and Toxicological Methods 47: 99-106, 2002.
35. Hileman, S. M., D.D. Pierroz, H. Masuzaki, C. Bjørbæk, K. El-Haschimi, W. A. Banks, and J. S. Flier. Characterizaton of Short Isoforms of the Leptin Receptor in Rat Cerebral Microvessels and of Brain Uptake of Leptin in Mouse Models of Obesity. Endocrinology 143:775-783, 2002.
36. Ishii, M., H. Fei, and J. M. Friedman. Targeted disruption of GPR7, the endogenous receptor for neuropeptides B and W, leads to metabolic defects and adult-onset obesity. PNAS 100:18:10540-10545, 2003.
37. Joseph, J. W., V. Koshkin, C-Y Zhang, J. Wang, B. B. Lowell, C. B. Chan, and M.B. Wheeler. Uncoupling Protein 2 Knockout Mice Have Enhanced Insulin Secretory Capacity After a High-Fat Diet. Diabetes 51:3211-3219, 2002.
38. Kim, S., et al. Effects of High-Fat, Angiotensinogen (agt) Gene Inactivation, and Targeted Expression to Adipose Tissue on Lipid Metabolism and Renal Gene Expression. Horm Metab Res 34:721-725, 2002.
39. Kumar, M. V., T. Shimokawa, T. R. Nagy, and M. D. Lane. Differential effects of a centrally acting fatty acid synthase inhibitor in lean and obese mice. PNAS 99:4: 1921-1925, 2002.
40. Lambert, P. D., et al. Ciliary neurotrophic factor activates leptin-like pathways and reduces body fat, without cachexia or rebound weight gain, even in leptin-resistant obesity. PNAS 98:8: 4652-4657, 2001.
41. Le Laya, S., et al. Decreased Resistin Expression in Mice with Different Sensitivities to a High-Fat Diet. Biochemical and Biophysical Research Communications 289:2:564-567, 2001.
42. Li, J., K. Takaishi, W. Cook, S. K. McCorkle, and R. H. Unger. Insig-1 "brakes" lipogenesis in adipocytes and inhibits differentiation of preadipocytes. PNAS 100:16:9476-9481, 2003.
43. Ludwig, D. S., et al. Melanin-concentrating hormone overexpression in transgenic mice leads to obesity and insulin resistance. J. Clin. Invest. 107:379-386, 2001.
44. Moon, Y. S., et al. Mice Lacking Paternally Expressed Pref-1/Dlk1 Display Growth Retardation and Accelerated Adiposity. Molecular And Cellular Biology 22:15: 5585-5592, 2002.
45. Murray, I., A. D. Sniderman, P. J. Havel, and K. Cianflonel. Acylation Stimulating Protein(ASP) Deficiency Alters Postprandial and Adipose Tissue Metabolism in Male Mice. The Journal Of Biological Chemistry 274:51:36219-36225, 1999.
46. Phan,L. K., F. Lin, C. A. LeDuc, W. K. Chung, and R. L. Leibel. The mouse mahoganoid coat color mutation disrupts a novel C3HC4 RING domain protein. J. Clin. Invest. 110:1449-1459, 2002.
47. Pierroz, D. D., M. Ziotopoulou, L. Ungsunan, S. Moschos, J. S. Flier, and C. S. Mantzoros. Effects of Acute and Chronic Administration of the Melanocortin Agonist MTII in Mice With Diet-Induced Obesity. Diabetes 51:1337-1345, 2002.
48. Rosenfeld, C. S., K. M. Grimm, K. A. Livingston, A. M. Brokman, W. E. Lamberson, and R. M. Roberts. Striking variation in the sex ratio of pups born to mice according to whether maternal diet is high in fat or carbohydrate. PNAS 100:8:4628-4632, 2003.
49. Chirala, S. S., et al. Fatty acid synthesis is essential in embryonic development: Fatty acid synthase null mutants and most of the heterozygotes die in utero. PNAS 100:11:6358-6363, 2003.
50. Takahashi, N., et al. Divergent Effects of Leptin in Mice Susceptible or Resistant to Obesity. Horm Metab Res 34:691-697, 2002.
51. Thupari, J. N., L. E. Landree, G. V. Ronnett, and F. P. Kuhajda. C75 increases peripheral energy utilization and fatty acid oxidation in diet-induced obesity. PNAS 99:14:9498-9502, 2002.
52. Tortoriello, D. V., J. Mcminn, and S. C. Chua. Dietary-Induced Obesity and Hypothalamic Infertility in Female DBA/2J Mice. Endocrinology 145: 1238-1247, 2004.
53. Weisberg, S. P., et al. Obesity is associated with macrophage accumulation in adipose tissue. J. Clin. Invest. 112:1796-1808, 2003.
54. Xu, H. et al. Chronic inflammation in fat plays a crucial role in the development of obesityrelated insulin resistance. J. Clin. Invest. 112:1821-1830, 2003.
55. Yamashita, T., et al. Enhanced insulin sensitivity in mice lacking ganglioside GM3. PNAS 100:6:3445-3449, 2003.
56. Bagnasco, M., M. G. Dube, A. Katz, P. S. Kalra, and S. P. Kalra. Leptin Expression In Hypothalamic Pvn Reverses Dietary Obesity And Hyperinsulinemia But Stimulates Ghrelin. Obes Res. 11:1463-1470, 2003.
57. Borowsky, B., et al. Antidepressant, anxiolytic and anorectic effects of a melaninconcentrating hormone-1 receptor antagonist. Nature Medicine 8:8:825-830, 2002.
58. Chan, C. B., et al. Increased Uncoupling Protein-2 Levels in b-cells Are Associated With Impaired Glucose-Stimulated Insulin Secretion. Diabetes 50:1302-1310, 2001.
59. Chen, L. and B. L. G. Nyomba. Glucose Intolerance and Resistin Expression in Rat Offspring Exposed to Ethanol in Utero: Modulation by Postnatal High-Fat Diet. Endocrinology 144:500-508, 2003.
60. Farley, C., J. A. Cook, B. D. Spar, T. M. Austin, and T. J. Kowalski. Meal Pattern Analysis Of Diet-Induced Obesity In Susceptible And Resistant Rats. Obes Res. 11:845-851, 2003.
61. Gao, J., L. Ghibaudi, M. Van Heek, J. J. Hwa. Characterization of diet-induced obese rats that develop persistent obesity after 6 months of high-fat followed by 1 month of low-fat diet. Brain Research 936:87-90, 2002.
62. Ogilvie, K. M., R. Saladin, T. R. Nagy, M. S. Urcan, R. A. Heyman, and M. D. Leibowitz. Activation of the Retinoid X Receptor Suppresses Appetite in the Rat. Endocrinology 145:565-573, 2004.
63. Shklyaev, S., et al. Sustained peripheral expression of transgene adiponectin offsets the development of diet-induced obesity in rats. PNAS 100:24:14217-14222, 2003.
64. Hildebrandt, A. L., D. M. Kelly-Sullivan, S. C. Black. Antiobesity effects of chronic cannabinoid CB1 receptor antagonist treatment in diet-induced obese mice. European Journal of Pharmacology 462:125-132, 2003.

Open formula purified diets for lab animals

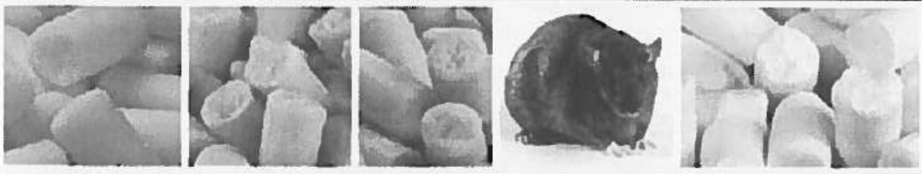

OpenSource DIETS®

Product Data - D12451

Report ▶ Repeat ▶ Revise

Description
Rodent Diet with 45% kcal% fat.

Used in Research
Fatty Liver
Inflammation
Obesity
Diabetes

Packaging
Product is packed in 12.5 kg box. Each box is identified with the product name, description, lot number and expiration date.

Lead Time
IN-STOCK.
Ready for next day shipment.

Gamma-Irradiation
Yes. Add 10 days to delivery time.

Form
Pellet, Powder, Liquid

Shelf Life
Most diets require storage in a cool dry environment. Stored correctly they should last 6 months.

Control Diets
D12450B, D12450H, D12450K

Formula

Product #D12451	gm%	kcal%
Protein	24	20
Carbohydrate	41	35
Fat	24	45
Total		100
kcal/gm	4.73	

Ingredient	gm	kcal
Casein, 30 Mesh	200	800
L-Cystine	3	12
Corn Starch	72.8	291
Maltodextrin 10	100	400
Sucrose	172.8	691
Cellulose, BW200	50	0
Soybean Oil	25	225
Lard*	177.5	1598
Mineral Mix S10026	10	0
DiCalcium Phosphate	13	0
Calcium Carbonate	5.5	0
Potassium Citrate, 1 H2O	16.5	0
Vitamin Mix V10001	10	40
Choline Bitartrate	2	0
FD&C Red Dye #40	0.05	0
**Total**	**858.15**	**4057**

Formulated by E. A. Ulman, Ph.D., Research Diets, Inc., 8/26/98 and 3/11/99.

*Typical analysis of cholesterol in lard = 0.72 mg/gram.
Cholesterol (mg)/4057 kcal = 167.8
Cholesterol (mg)/kg = 195.5

RESEARCH DIETS INC.
www.ResearchDiets.com
Where NutriPhenomics Begins

Research Diets, Inc.
20 Jules Lane
New Brunswick, NJ 08901 USA
Tel: 732.247.2390
Fax: 732.247.2340
info@researchdiets.com

© 2006 Research Diets, Inc. All rights reserved. D12451-2-13

D12450B, D12451, D12492 The "Original High Fat Diets"

1. Van Heek M, et al. Diet-induced Obese Mice Develop Peripheral, but not Central Resistance to Leptin. J. Clin. Invest. 1997; 99:385-390.
2. Taheri S, et al. Distribution and quantification of immunoreactive orexin A in rat tissues. FEBS Lett. 1999 Aug 20; 457(1):157-61.
3. Nonogaki K, et al. Leptin-independent hyperphagia and type 2 diabetes in mice with a mutated serotonin 5-HT2C receptor gene. Nature Medicine 1998; 4:1152-1156.
4. El-Haschimi K, et al. Two defects contribute to hypothalamic leptin resistance in mice with diet-induced obesity. J.Clin.Invest. 2000; 105:1827-1832.
5. Bush EN, et al.(Metabolic Disease Research, Abbott Laboratories) Adiposity, Leptin Resistance, Hyperrphagia, Hyperglycemia, Glucose Intolerance and Insulin Resistance in C57BL/6J Mice Fed High Fat Diets.Endocrine SocietyAnnual Meeting 2001, Poster Session.
6. Shapiro ME, et al. (Metabolic Disease Research, Abbott Laboratories) Effects of Treatment of C57BL/6j Mice Fed High vs. Low Fat Diets with Metformin or Rosiglitazone on Adiposity, Food Intake, Hyperglycemia and Insulin Resistance. Endocrine Society Annual Meeting 2001, Poster Session.
7. Masuzaki H, Paterson J, Shinyama H, Morton NM, Mullins JJ, Seckl JR, Flier JS. A Transgenic Model of Visceral Obesity and the Metabolic Syndrome. Science. 2001; 294:2166-2170.
8. Razani B, Combs TP, Wang XB, Frank PG, Park DS, Russell RG, Li M, Tang B, Jelicks LA, Scherer PE, Lisanti MP. Caveolin-1 deficient mice are lean, resistant to diet-induced obesity, and show hyper-triglyceridemia with adipocyte abnormalities. J. Biol. Chem. 2001; (in press).
9. Valet P, Grujic D, Wade J, Ito M, Zingaretti MC, Soloveva V, Ross SR, Graves RA, Cinti S, Lafontan M, and Lowell BB. Expression of human alpha 2-adrenergic receptors in adipose tissue of beta 3-adrenergic receptor-deficient mice promotes diet-induced obesity. J.Biol.Chem. 275: 34797-34802, 2000.
10. Vidal-Puig AJ, Grujic D, Zhang CY, Hagen T, Boss O, Ido Y, Szczepanik A, Wade J, Mootha V, Cortright R, Muoio DM, and Lowell BB. Energy metabolism in uncoupling protein 3 gene knockout mice. J.Biol.Chem. 275: 16258-16266, 2000.
11. Ziotopoulou M, Mantzoros CS, Hileman SM, & Flier JS. Differential expression of hypothalamic neuropeptides in the early phase of diet-induced obesity in mice. Am. J. Physiol. Endocrinol. Metab. 279:E383-E845, 2000.
12. Scrocchi LA & Drucker DJ. Effects of Aging and a High Fat Diet on Body Weight and Glucose Tolerance in Glucagon-Like Peptide-1 Receptor -/- Mice. Endocrinology 139:3127- 3132, 1998.
13. Ghibaudi L, Cook J, Farley C, Van Heek M, & Hwa J.Fat Intake Affects Adiposity, Comorbidity Factors, and Energy Metabolism of Sprague-Dawley Rats. Obes. Res. 10:956-963. 2002
14. Dube MG, Beretta E, Dhillon H, Ueno N, Kalra PS & Kalra SP. Central Leptin Gene Therapy Blocks High-Fat Diet-Induced Weight Gain, Hyperleptinemeia, and Hyperinsulinemia. Diabetes 51:1729-1736, 2001.
15. Tang H, Vasselli JR, Wu EX, Boozer CN, & Gallagher D. High-Resolution Magnetic Resonance Imaging Tracks Changes in Organ and Tissue Mass in Obese and Aging Rats. Am J Physiol (Regulatory Integrative Comp Physiol) 282:R890-R899, 2002.
16. Bowen H, Mitchell TD, & Harris RBS (Dept. of Foods and Nutr, U of Georgia). Method of Leptin Dosing, Strain, and Group Housing Influence Leptin Sensitivity in High-Fet-Fed Weanling Mice. AJP-Regul Integr Comp Physiol 284:R87-R100, 2003.
17. Harris RBS, Mitchell TD, & Hebert S (Pennington Biomedical Research Center, Baton Rouge, LA). Leptin-Induced Changes in Body Composition in High Fat-Fed Mice. AJPRegul Integr Comp Physiol 284:R87-R100, 2003.
18. Anini, Y. and Brubaker, P. L. Role of Leptin in the Regulation of Glucagon-Like Peptide-1 Secretion. Diabetes 52:252-259, 2003.
19. Bluher, S., Responsiveness to Peripherally Administered Melanocortins in Lean and Obese Mice. Diabetes 53:82-90, 2004.
20. Brunengraber, D. Z., et al. Influence of diet on the modeling of adipose tissue triglycerides during growth. Am J Physiol Endocrinol Metab 285: E917-E925, 2003.
21. Challis, B. G., et al. Mice lacking pro-opiomelanocortin are sensitive to high-fat feeding but respond normally to the acute anorectic effects of peptide-YY3-36. PNAS 101: 13: 4695-4700, 2004.
22. Cohen, A. W., B. Razani, X. B. Wang, T. P. Combs, T. M. Williams, P. E. Scherer, and M. P. Lisanti. Caveolin-1-deficient mice show insulin resistance and defective insulin receptor protein expression in adipose tissue. Am J Physiol Cell Physiol 285: C222-C235, 2003.
23. Combs, T. P., et al. A Transgenic Mouse with a Deletion in the Collagenous Domain of Adiponectin Displays Elevated Circulating Adiponectin and Improved Insulin Sensitivity. Endocrinology 145:367-383, 2004.
24. Conarello, S. L., et al. Mice lacking dipeptidyl peptidase IV are protected against obesity and insulin resistance. PNAS, 100:11: 6825-6830, 2003.
25. Dhar, M. S., et al. Mice Heterozygous for Atp10c, a Putative Amphipath, Represent a Novel Model of Obesity and Type 2 Diabetes. J. Nutr. 134: 799-805, 2004.
26. El-Haschimi, K., S. D. Dufresne, M. F. Hirshman, J. S. Flier, L. J. Goodyear, and C. Bjørbæk. Insulin Resistance and Lipodystrophy in Mice Lacking Ribosomal S6 Kinase 2. Diabetes 52:1340-1346, 2003.
27. Felipe, F., M. L. Bonet, J. Ribot, and A. Palou. Modulation of Resistin Expression by Retinoic Acid and Vitamin A Status. Diabetes 53:882-889, 2004.
28. Felipe, F., M.L. Bonet, J. Ribot and A. Palou. Up-regulation of muscle uncoupling protein 3 gene expression in mice following high fat diet, dietary vitamin A supplementation and acute retinoic acid-treatment. International Journal of Obesity 27: 60-69, 2003.
29. Fu, J., et al. Oleylethanolamide regulates feeding and body weight through activation of the nuclear receptor PPAR. Nature 425:90-93, 2003.
30. Gavrilova, O., et al. Liver Peroxisome Proliferator-activated Receptor Contributes to Hepatic Steatosis, Triglyceride Clearance, and Regulation of Body Fat Mass. The Journal of Biological Chemistry 278:36:34268-34276, 2003.
31. Haluzik, M., O. Gavrilova and D. LeRoith. Peroxisome Proliferator-Activated Receptor-Deficiency Does Not Alter Insulin Sensitivity in Mice Maintained on Regular or High-Fat Diet: Hyperinsulinemic-Euglycemic Clamp Studies. Endocrinology 145:1662-1667, 2004
32. Hancock, A.A. et al. Antiobesity effects of A-331440, a novel non-imidazole histamine
32. Hancock, A.A. et al. Antiobesity effects of A-331440, a novel non-imidazole histamine H3 receptor antagonist. European Journal of Pharmacology 487:183-197, 2004.
33. Hennige, A. M., et al. Upregulation of insulin receptor substrate-2 in pancreatic b cells prevents diabetes. J. Clin. Invest. 112:1521-1532, 2003.34.
34. Hildebrandt, A. L., D. M. Kelly-Sullivan, S. C. Black. Validation of a high-resolution X-ray computed tomography system to measure murine adipose tissue depot mass in situ and longitudinally. Journal of Pharmacological and Toxicological Methods 47: 99- 106, 2002.
35. Hileman, S. M., D.D. Pierroz, H. Masuzaki, C. Bjørbæk, K. El-Haschimi, W. A. Banks, and J. S. Flier. Characterizaton of Short Isoforms of the Leptin Receptor in Rat Cerebral Microvessels and of Brain Uptake of Leptin in Mouse Models of Obesity. Endocrinology 143:775-783, 2002.
36. Ishii, M., H. Fei, and J. M. Friedman. Targeted disruption of GPR7, the endogenous receptor for neuropeptides B and W, leads to metabolic defects and adult-onset obesity. PNAS 100:18:10540-10545, 2003.
37. Joseph, J. W., V. Koshkin, C-Y Zhang, J. Wang, B. B. Lowell, C. B. Chan, and M.B. Wheeler. Uncoupling Protein 2 Knockout Mice Have Enhanced Insulin Secretory Capacity After a High-Fat Diet. Diabetes 51:3211-3219, 2002.
38. Kim, S., et al. Effects of High-Fat, Angiotensinogen (agt) Gene Inactivation, and Targeted Expression to Adipose Tissue on Lipid Metabolism and Renal Gene Expression. Horm Metab Res 34:721-725, 2002.
39. Kumar, M. V., T. Shimokawa, T. R. Nagy, and M. D. Lane. Differential effects of a centrally acting fatty acid synthase inhibitor in lean and obese mice. PNAS 99:4: 1921-1925, 2002.
40. Lambert, P. D., et al. Ciliary neurotrophic factor activates leptin-like pathways and reduces body fat, without cachexia or rebound weight gain, even in leptin-resistant obesity. PNAS 98:8: 4652-4657, 2001.
41. Le Laya, S., et al. Decreased Resistin Expression in Mice with Different Sensitivities to a High-Fat Diet. Biochemical and Biophysical Research Communications 289:2:564-567, 2001.
42. Li, J., K. Takaishi, W. Cook, S. K. McCorkle, and R. H. Unger. Insig-1 "brakes" lipogenesis in adipocytes and inhibits differentiation of preadipocytes. PNAS 100:16:9476-9481, 2003.
43. Ludwig, D. S., et al. Melanin-concentrating hormone overexpression in transgenic mice leads to obesity and insulin resistance. J. Clin. Invest. 107:379-386, 2001.
44. Moon, Y. S., et al. Mice Lacking Paternally Expressed Pref-1/Dlk1 Display Growth Retardation and Accelerated Adiposity. Molecular And Cellular Biology 22:15: 5585-5592, 2002.
45. Murray, I., A. D. Sniderman, P. J. Havel, and K. Cianflonel. Acylation Stimulating Protein (ASP) Deficiency Alters Postprandial and Adipose Tissue Metabolism in Male Mice. The Journal Of Biological Chemistry 274:51:36219-36225, 1999.
46. Phan,L. K., F. Lin, C. A. LeDuc, W. K. Chung, and R. L. Leibel. The mouse " mahoganoid coat color mutation disrupts a novel C3HC4 RING domain protein. J. Clin. Invest. 110:1449-1459, 2002.
47. Pierroz, D. D., M. Ziotopoulou, L. Ungsunan, S. Moschos, J. S. Flier, and C. S. Mantzoros. Effects of Acute and Chronic Administration of the Melanocortin Agonist MTII in Mice With Diet-Induced Obesity. Diabetes 51:1337-1345, 2002.
48. Rosenfeld, C. S., K. M. Grimm, K. A. Livingston, A. M. Brokman, W. E. Lamberson, and R. M. Roberts. Striking variation in the sex ratio of pups born to mice according to whether maternal diet is high in fat or carbohydrate. PNAS 100:8:4628-4632, 2003.
49. Chirala, S. S., et al. Fatty acid synthesis is essential in embryonic development: Fatty acid synthase null mutants and most of the heterozygotes die in utero. PNAS 100:11:6358-6363, 2003.
50. Takahashi, N., et al. Divergent Effects of Leptin in Mice Susceptible or Resistant to Obesity. Horm Metab Res 34:691-697, 2002.
51. Thupari, J. N., L. E. Landree, G. V. Ronnett, and F. P. Kuhajda. C75 increases peripheral energy utilization and fatty acid oxidation in diet-induced obesity. PNAS 99:14:9498-9502, 2002.
52. Tortoriello, D. V., J. Mcminn, and S. C. Chua. Dietary-Induced Obesity and Hypothalamic Infertility in Female DBA/2J Mice. Endocrinology 145: 1238-1247, 2004.
53. Weisberg, S. P., et al. Obesity is associated with macrophage accumulation in adipose tissue. J. Clin. Invest. 112:1796-1808, 2003.
54. Xu, H. et al. Chronic inflammation in fat plays a crucial role in the development of obesity related insulin resistance. J. Clin. Invest. 112:1821-1830, 2003.
55. Yamashita, T., et al. Enhanced insulin sensitivity in mice lacking ganglioside GM3. PNAS 100:6:3445-3449, 2003.
56. Bagnasco, M., M. G. Dube, A. Katz, P. S. Kalra, and S. P. Kalra. Leptin Expression In Hypothalamic Pvn Reverses Dietary Obesity And Hyperinsulinemia But Stimulates Ghrelin. Obes Res. 11:1463-1470, 2003.
57. Borowsky, B., et al. Antidepressant, anxiolytic and anorectic effects of a melaninconcentrating hormone-1 receptor antagonist. Nature Medicine 8:8:825-830, 2002.
58. Chan, C. B., et al. Increased Uncoupling Protein-2 Levels in b-cells Are Associated With Impaired Glucose-Stimulated Insulin Secretion. Diabetes 50:1302-1310, 2001.
59. Chen, L. and B. L. G. Nyomba. Glucose Intolerance and Resistin Expression in Rat Offspring Exposed to Ethanol in Utero: Modulation by Postnatal High-Fat Diet. Endocrinology 144:500-508, 2003.
60. Farley, C., J. A. Cook, B. D. Spar, T. M. Austin, and T. J. Kowalski. Meal Pattern Analysis Of Diet-Induced Obesity In Susceptible And Resistant Rats. Obes Res. 11:845-851, 2003.
61. Gao, J., L. Ghibaudi, M. Van Heek, J. J. Hwa. Characterization of diet-induced obese rats that develop persistent obesity after 6 months of high-fat followed by 1 month of low-fat diet. Brain Research 936:87-90, 2002.
62. Ogilvie, K. M., R. Saladin, T. R. Nagy, M. S. Urcan, R. A. Heyman, and M. D. Leibowitz. Activation of the Retinoid X Receptor Suppresses Appetite in the Rat. Endocrinology 145:565-573, 2004.
63. Shklyaev, S., et al. Sustained peripheral expression of transgene adiponectin offsets the development of diet-induced obesity in rats. PNAS 100:24:14217-14222, 2003.
64. Hildebrandt, A. L., D. M. Kelly-Sullivan, S. C. Black. Antiobesity effects of chronic cannabinoid CB1 receptor antagonist treatment in diet-induced obese mice. European Journal of Pharmacology 462:125-132, 2003.

Product Data

D12492

Description

Rodent Diet with 60% kcal% fat.

Used in Research

Obesity
Diabetes

Packaging

Product is packed in 12.5 kg box. Each box is identified with the product name, description, lot number and expiration date.

Lead Time

IN-STOCK. Ready for next day shipment.

Gamma-Irradiation

Yes. Add 10 days to delivery time.

Form

Pellet, Powder, Liquid

Shelf Life

Most diets require storage in a cool dry environment. Stored correctly they should last 3-6 months. Because of the high fat content is best if kept frozen.

Control Diets

D12450B

Formula

Product #	D12492	
	gm%	kcal%
Protein	26.2	20
Carbohydrate	26.3	20
Fat	34.9	60
Total		100
kcal/gm	5.24	

Ingredient	gm	kcal
Casein, 80 Mesh	200	800
L-Cystine	3	12
Corn Starch	0	0
Maltodextrin 10	125	500
Sucrose	68.8	275.2
Cellulose, BW200	50	0
Soybean Oil	25	225
Lard*	245	2205
Mineral Mix, S10026	10	0
DiCalcium Phosphate	13	0
Calcium Carbonate	5.5	0
Potassium Citrate, 1 H2O	16.5	0
Vitamin Mix, V10001	10	40
Choline Bitartrate	2	0
FD&C Blue Dye #1	0.05	0
Total	773.85	4057

Formulated by E. A. Ulman, Ph.D., Research Diets, Inc., 8/26/98 and 3/11/99.

*Typical analysis of cholesterol in lard = 0.95 mg/gram.
Cholesterol (mg)/4057 kcal = 232.8
Cholesterol (mg)/kg = 300.8

RESEARCH DIETS INC.
www.ResearchDiets.com

Research Diets, Inc.
20 Jules Lane
New Brunswick, NJ 08901
Tel: 732.247.2390
Fax: 732.247.2340
info@researchdiets.com

Copyright © 2006 Research Diets, Inc. All rights reserved. D12492

D12450B, D12451, D12492 The "Original High Fat Diets"

1. Van Heek M, et al. Diet-induced Obese Mice Develop Peripheral, but not Central Resistance to Leptin. J. Clin. Invest. 1997; 99:385-390.
2. Taheri S, et al. Distribution and quantification of immunoreactive orexin A in rat tissues. FEBS Lett. 1999 Aug 20; 457(1):157-61.
3. Nonogaki K, et al. Leptin-independent hyperphagia and type 2 diabetes in mice with a mutated serotonin 5-HT2C receptor gene. Nature Medicine 1998; 4:1152-1156.
4. El-Haschimi K, et al. Two defects contribute to hypothalamic leptin resistance in mice with diet-induced obesity. J.Clin.Invest. 2000; 105:1827-1832.
5. Bush EN, et al.(Metabolic Disease Research, Abbott Laboratories) Adiposity, Leptin Resistance, Hyperphagia, Hyperglycemia, Glucose Intolerance and Insulin Resistance in C57BL/6J Mice Fed High Fat Diets.Endocrine SocietyAnnual Meeting 2001, Poster Session.
6. Shapiro ME, et al. (Metabolic Disease Research, Abbott Laboratories) Effects of Treatment of C57BL/6j Mice Fed High vs. Low Fat Diets with Metformin or Rosiglitazone on Adiposity, Food Intake, Hyperglycemia and Insulin Resistance .Endocrine Society Annual Meeting 2001, Poster Session.
7. Masuzaki H, Paterson J, Shinyama H, Morton NM, Mullins JJ, Seckl JR, Flier JS. A Transgenic Model of Visceral Obesity and the Metabolic Syndrome. Science. 2001; 294:2166-2170.
8. Razani B, Combs TP, Wang XB, Frank PG, Park DS, Russell RG, Li M, Tang B, Jelicks LA, Scherer PE, Lisanti MP. Caveolin-1 deficient mice are lean, resistant to diet-induced obesity, and show hyper-triglyceridemia with adipocyte abnormalities. J. Biol. Chem. 2001; (in press).
9. Valet P, Grujic D, Wade J, Ito M, Zingaretti MC, Soloveva V, Ross SR, Graves RA, Cinti S, Lafontan M, and Lowell BB. Expression of human alpha 2-adrenergic receptors in adipose tissue of beta 3-adrenergic receptor-deficient mice promotes diet-induced obesity. J.Biol.Chem. 275: 34797-34802, 2000.
10. Vidal-Puig AJ, Grujic D, Zhang CY, Hagen T, Boss O, Ido Y, Szczepanik A, Wade J, Mootha V, Cortright R, Muoio DM, and Lowell BB. Energy metabolism in uncoupling protein 3 gene knockout mice. J.Biol.Chem. 275: 16258-16266, 2000.
11. Ziotopoulou M, Mantzoros CS, Hileman SM, & Flier JS. Differential expression of hypothalamic neuropeptides in the early phase of diet-induced obesity in mice. Am. J. Physiol. Endocrinol. Metab. 279:E383-E845, 2000.
12. Scrocchi LA & Drucker DJ. Effects of Aging and a High Fat Diet on Body Weight and Glucose Tolerance in Glucagon-Like Peptide-1 Receptor -/- Mice. Endocrinology 139:3127-3132, 1998.
13. Ghibaudi L, Cook J, Farley C, Van Heek M, & Hwa J.Fat Intake Affects Adiposity, Comorbidity Factors, and Energy Metabolism of Sprague-Dawley Rats. Obes. Res. 10:956-963. 2002
14. Dube MG, Beretta E, Dhillon H, Ueno N, Kalra PS & Kalra SP. Central Leptin Gene Therapy Blocks High-Fat Diet-Induced Weight Gain, Hyperleptinemeia, and Hyperinsulinemia. Diabetes 51:1729-1736, 2001.
15. Tang H, Vasselli JR, Wu EX, Boozer CN, & Gallagher D. High-Resolution Magnetic Resonance Imaging Tracks Changes in Organ and Tissue Mass in Obese and Aging Rats. Am J Physiol (Regulatory Integrative Comp Physiol) 282:R890-R899, 2002.
16. Bowen H, Mitchell TD, & Harris RBS (Dept. of Foods and Nutr, U of Georgia). Method of Leptin Dosing, Strain, and Group Housing Influence Leptin Sensitivity in High-Fet-Fed Weanling Mice. AJP-Regul Integr Comp Physiol 284:R87-R100, 2003.
17. Harris RBS, Mitchell TD, & Hebert S (Pennington Biomedical Research Center, Baton Rouge, LA). Leptin-Induced Changes in Body Composition in High Fat-Fed Mice. AJP-Regul Integr Comp Physiol 284:R87-R100, 2003.
18. Anini, Y. and Brubaker, P. L. Role of Leptin in the Regulation of Glucagon-Like Peptide-1 Secretion. Diabetes 52:252-259, 2003.
19. Bluher, S., Responsiveness to Peripherally Administered Melanocortins in Lean and Obese Mice. Diabetes 53:82-90, 2004.
20. Brunengraber, D. Z., et al. Influence of diet on the modeling of adipose tissue triglycerides during growth. Am J Physiol Endocrinol Metab 285: E917-E925, 2003.
21. Challis, B. G., et al. Mice lacking pro-opiomelanocortin are sensitive to high-fat feeding but respond normally to the acute anorectic effects of peptide-YY3-36. PNAS 101: 13: 4695-4700, 2004.
22. Cohen, A. W., B. Razani, X. B. Wang, T. P. Combs, T. M. Williams, P. E. Scherer, and M. P. Lisanti. Caveolin-1-deficient mice show insulin resistance and defective insulin receptor protein expression in adipose tissue. Am J Physiol Cell Physiol 285: C222-C235, 2003.
23. Combs, T. P., et al. A Transgenic Mouse with a Deletion in the Collagenous Domain of Adiponectin Displays Elevated Circulating Adiponectin and Improved Insulin Sensitivity. Endocrinology 145:367-383, 2004.
24. Conarello, S. L., et al. Mice lacking dipeptidyl peptidase IV are protected against obesity and insulin resistance. PNAS, 100:11: 6825-6830, 2003.
25. Dhar, M. S., et al. Mice Heterozygous for Atp10c, a Putative Amphipath, Represent a Novel Model of Obesity and Type 2 Diabetes. J. Nutr. 134: 799-805, 2004.
26. El-Haschimi, K., S. D. Dufresne, M. F. Hirshman, J. S. Flier, L. J. Goodyear, and C. Bjørbæk. Insulin Resistance and Lipodystrophy in Mice Lacking Ribosomal S6 Kinase 2. Diabetes 52:1340-1346, 2003.
27. Felipe, F., M. L. Bonet, J. Ribot, and A. Palou. Modulation of Resistin Expression by Retinoic Acid and Vitamin A Status. Diabetes 53:882-889, 2004.
28. Felipe, F., M.L. Bonet, J. Ribot and A. Palou. Up-regulation of muscle uncoupling protein 3 gene expression in mice following high fat diet, dietary vitamin A supplementation and acute retinoic acid-treatment. International Journal of Obesity 27: 60-69, 2003.
29. Fu, J., et al. Oleylethanolamide regulates feeding and body weight through activation of the nuclear receptor PPAR. Nature 425:90-93, 2003.
30. Gavrilova, O., et al. Liver Peroxisome Proliferator-activated Receptor Contributes to Hepatic Steatosis, Triglyceride Clearance, and Regulation of Body Fat Mass. The Journal of Biological Chemistry 278:36:34268-34276, 2003.
31. Haluzik, M., O. Gavrilova and D. LeRoith. Peroxisome Proliferator-Activated Receptor-Deficiency Does Not Alter Insulin Sensitivity in Mice Maintained on Regular or High-Fat Diet: Hyperinsulinemic-Euglycemic Clamp Studies. Endocrinology 145:1662-1667, 2004.
32. Hancock, A.A. et al. Antiobesity effects of A-331440, a novel non-imidazole histamine H3 receptor antagonist. European Journal of Pharmacology 487:183-197, 2004.
33. Hennige, A. M., et al. Upregulation of insulin receptor substrate-2 in pancreatic b cells prevents diabetes. J. Clin. Invest. 112:1521-1532, 2003.34.
34. Hildebrandt, A. L., D. M. Kelly-Sullivan, S. C. Black. Validation of a high-resolution X-ray computed tomography system to measure murine adipose tissue depot mass in situ and longitudinally. Journal of Pharmacological and Toxicological Methods 47: 99- 106, 2002.
35. Hileman, S. M., D.D. Pierroz, H. Masuzaki, C. Bjørbæk, K. El-Haschimi, W. A. Banks, and J. S. Flier. Characterizaton of Short Isoforms of the Leptin Receptor in Rat Cerebral Microvessels and of Brain Uptake of Leptin in Mouse Models of Obesity. Endocrinology 143:775-783, 2002.
36. Ishii, M., H. Fei, and J. M. Friedman. Targeted disruption of GPR7, the endogenous receptor for neuropeptides B and W, leads to metabolic defects and adult-onset obesity. PNAS 100:18:10540-10545, 2003.
37. Joseph, J. W., V. Koshkin, C-Y Zhang, J. Wang, B. B. Lowell, C. B. Chan, and M.B. Wheeler. Uncoupling Protein 2 Knockout Mice Have Enhanced Insulin Secretory Capacity After a High-Fat Diet. Diabetes 51:3211-3219, 2002.
38. Kim, S., et al. Effects of High-Fat, Angiotensinogen (agt) Gene Inactivation, and Targeted Expression to Adipose Tissue on Lipid Metabolism and Renal Gene Expression. Horm Metab Res 34:721-725, 2002.
39. Kumar, M. V., T. Shimokawa, T. R. Nagy, and M. D. Lane. Differential effects of a centrally acting fatty acid synthase inhibitor in lean and obese mice. PNAS 99:4: 1921-1925, 2002.
40. Lambert, P. D., et al. Ciliary neurotrophic factor activates leptin-like pathways and reduces body fat, without cachexia or rebound weight gain, even in leptin-resistant obesity. PNAS 98:8: 4652-4657, 2001.
41. Le Laya, S., et al. Decreased Resistin Expression in Mice with Different Sensitivities to a High-Fat Diet. Biochemical and Biophysical Research Communications 289:2:564-567, 2001.
42. Li, J., K. Takaishi, W. Cook, S. K. McCorkle, and R. H. Unger. Insig-1 "brakes" lipogenesis in adipocytes and inhibits differentiation of preadipocytes. PNAS 100:16:9476-9481, 2003.
43. Ludwig, D. S., et al. Melanin-concentrating hormone overexpression in transgenic mice leads to obesity and insulin resistance. J. Clin. Invest. 107:379-386, 2001.
44. Moon, Y. S., et al. Mice Lacking Paternally Expressed Pref-1/Dlk1 Display Growth Retardation and Accelerated Adiposity. Molecular And Cellular Biology 22:15: 5585-5592, 2002.
45. Murray, I., A. D. Sniderman, P. J. Havel, and K. Cianflonei. Acylation Stimulating Protein (ASP) Deficiency Alters Postprandial and Adipose Tissue Metabolism in Male Mice. The Journal Of Biological Chemistry 274:51:36219-36225, 1999.
46. Phan,L. K., F. Lin, C. A. LeDuc, W. K. Chung, and R. L. Leibel. The mouse mahoganoid coat color mutation disrupts a novel C3HC4 RING domain protein. J. Clin. Invest. 110:1449-1459, 2002.
47. Pierroz, D. D., M. Ziotopoulou, L. Ungsunan, S. Moschos, J. S. Flier, and C. S. Mantzoros. Effects of Acute and Chronic Administration of the Melanocortin Agonist MTII in Mice With Diet-Induced Obesity. Diabetes 51:1337-1345, 2002.
48. Rosenfeld, C. S., K. M. Grimm, K. A. Livingston, A. M. Brokman, W. E. Lamberson, and R. M. Roberts. Striking variation in the sex ratio of pups born to mice according to whether maternal diet is high in fat or carbohydrate. PNAS 100:8:4628-4632, 2003.
49. Chirala, S. S., et al. Fatty acid synthesis is essential in embryonic development: Fatty acid synthase null mutants and most of the heterozygotes die in utero. PNAS 100:11:6358-6363, 2003.
50. Takahashi, N., et al. Divergent Effects of Leptin in Mice Susceptible or Resistant to Obesity. Horm Metab Res 34:691-697, 2002.
51. Thupari, J. N., L. E. Landree, G. V. Ronnett, and F. P. Kuhajda. C75 increases peripheral energy utilization and fatty acid oxidation in diet-induced obesity. PNAS 99:14:9498-9502, 2002.
52. Tortoriello, D. V., J. Mcminn, and S. C. Chua. Dietary-Induced Obesity and Hypothalamic Infertility in Female DBA/2J Mice. Endocrinology 145: 1238-1247, 2004.
53. Weisberg, S. P., et al. Obesity is associated with macrophage accumulation in adipose tissue. J. Clin. Invest. 112:1796-1808, 2003.
54. Xu, H. et al. Chronic inflammation in fat plays a crucial role in the development of obesity-related insulin resistance. J. Clin. Invest. 112:1821-1830, 2003.
55. Yamashita, T., et al. Enhanced insulin sensitivity in mice lacking ganglioside GM3. PNAS 100:6:3445-3449, 2003.
56. Bagnasco, M., M. G. Dube, A. Katz, P. S. Kalra, and S. P. Kalra. Leptin Expression In Hypothalamic Pvn Reverses Dietary Obesity And Hyperinsulinemia But Stimulates Ghrelin. Obes Res. 11:1463-1470, 2003.
57. Borowsky, B., et al. Antidepressant, anxiolytic and anorectic effects of a melanin-concentrating hormone-1 receptor antagonist. Nature Medicine 8:8:825-830, 2002.
58. Chan, C. B., et al. Increased Uncoupling Protein-2 Levels in b-cells Are Associated With Impaired Glucose-Stimulated Insulin Secretion. Diabetes 50:1302-1310, 2001.
59. Chen, L. and B. L. G. Nyomba. Glucose Intolerance and Resistin Expression in Rat Offspring Exposed to Ethanol in Utero: Modulation by Postnatal High-Fat Diet. Endocrinology 144:500-508, 2003.
60. Farley, C., J. A. Cook, B. D. Spar, T. M. Austin, and T. J. Kowalski. Meal Pattern Analysis Of Diet-Induced Obesity In Susceptible And Resistant Rats. Obes Res. 11:845-851, 2003.
61. Gao, J., L. Ghibaudi, M. Van Heek, J. J. Hwa. Characterization of diet-induced obese rats that develop persistent obesity after 6 months of high-fat followed by 1 month of low-fat diet. Brain Research 936:87-90, 2002.
62. Ogilvie, K. M., R. Saladin, T. R. Nagy, M. S. Urcan, R. A. Heyman, and M. D. Leibowitz. Activation of the Retinoid X Receptor Suppresses Appetite in the Rat. Endocrinology 145:565-573, 2004.
63. Shklyaev, S., et al. Sustained peripheral expression of transgene adiponectin offsets the development of diet-induced obesity in rats. PNAS 100:24:14217-14222, 2003.
64. Hildebrandt, A. L., D. M. Kelly-Sullivan, S. C. Black. Antiobesity effects of chronic cannabinoid CB1 receptor antagonist treatment in diet-induced obese mice. European Journal of Pharmacology 462:125-132, 2003.

abdiets | Product information

High Fat diet with Lard 4031,09

Purified diet for rat and mice

Analysis

Crude protein	%	21,40
Crude fat	%	23,50
Crude fibre	%	6,00
Ash	%	2,20
Dry matter	%	93,70
Starch	%	16,00
Sugar	%	19,00

Energy

GE	kcal/g	5,3
ME	kcal/g	4,6

Minerals

Ca	%	0,86
P	%	0,52
K	%	0,70
Mg	%	0,20
Na	%	0,14
Cl	%	0,67

Trace elements, total

Fe	mg/kg	105,00
Mn	mg/kg	70,00
Zn	mg/kg	55,00
Cu	mg/kg	17,50
Co	mg/kg	0,20
J	mg/kg	0,40

Amino acids

Lysine	g/kg	14,90
Methionine	g/kg	8,70
Meth+Cys	g/kg	9,30
Cystine	g/kg	0,60
Threonine	g/kg	8,50
Tryptofaan	g/kg	2,80
Isoleucine	g/kg	12,10
Arginine	g/kg	7,30
Fenylalanine	g/kg	8,70
Histidine	g/kg	5,60
Leucine	g/kg	22,80
Tyrosine	g/kg	11,30
Valine	g/kg	14,10

Vitamins, added

Vit.A	IU/kg	21600,00
Vit.D3	IU/kg	2400,00
Vit.E	mg/kg	75,00
Vit.K3	mg/kg	12,00
B1 thiam	mg/kg	24,00
B2 ribof	mg/kg	14,00
B6 pyrid	mg/kg	18,00
B3 Nicot	mg/kg	47,00
B5 Panto	mg/kg	19,00
Vit.B12	µg/kg	60,00
B9 Folin	mg/kg	9,40
Choline	mg/kg	1490,00
Biotine	µg/kg	368,00
Vit.C	mg/kg	0,00

Linoleic	g/kg	96,00

abdiets | Product information

AM-II 2141

Standard diet for the breeding and the maintenance of rats, mice and hamsters

Analysis

Crude protein	%	25,50
Crude fat	%	6,50
Crude fibre	%	3,50
Ash	%	5,00
Dry matter	%	91,00
Starch	%	38,80
Sugar	%	4,60

Energy

GE	kJ/kg	17500

Minerals

Ca	%	0,90
P	%	0,55
K	%	0,85
Mg	%	0,12
Na	%	0,25
Cl	%	0,60

Trace elements, total

Fe	mg/kg	130,00
Mn	mg/kg	55,00
Zn	mg/kg	77,00
Cu	mg/kg	20,00
Co	mg/kg	1,60
J	mg/kg	0,20

Amino acids

Lysine	g/kg	12,00
Methionine	g/kg	4,00
Meth+Cys	g/kg	7,30
Cystine	g/kg	3,30
Threonine	g/kg	8,50
Tryptofaan	g/kg	2,50
Isoleucine	g/kg	8,30
Arginine	g/kg	15,60
Fenylalanine	g/kg	9,80
Histidine	g/kg	5,00
Leucine	g/kg	12,00
Tyrosine	g/kg	4,50
Valine	g/kg	11,70

Vitamins, added

Vit.A	IU/kg	16000,00
Vit.D3	IU/kg	1500,00
Vit.E	mg/kg	88,00
Vit.K3	mg/kg	2,50
B1 thiam	mg/kg	15,60
B2 ribof	mg/kg	10,00
B6 pyrid	mg/kg	14,00
B3 Nicot	mg/kg	40,80
B5 Panto	mg/kg	22,00
Vit.B12	µg/kg	55,00
B9 Folin	mg/kg	4,00
Choline	mg/kg	140,00
Biotine	µg/kg	500,00

Linoleic acid	g/kg	18,50
